# Optical Imaging of Paramagnetic Bead-DNA Aggregation Inhibition Allows for Low Copy Number Detection of Infectious Pathogens

**DOI:** 10.1371/journal.pone.0129830

**Published:** 2015-06-11

**Authors:** Jacquelyn A. DuVall, Juliane C. Borba, Nazly Shafagati, Deborah Luzader, Nishant Shukla, Jingyi Li, Kylene Kehn-Hall, Melissa M. Kendall, Sanford H. Feldman, James P. Landers

**Affiliations:** 1 Department of Chemistry, University of Virginia, Charlottesville, VA, United States of America; 2 National Center for Biodefense and Infectious Diseases, George Mason University, Manassas, VA, United States of America; 3 Department of Microbiology, Immunology, and Cancer Biology, University of Virginia, Charlottesville, VA, United States of America; 4 Department of Computer Science, University of Virginia, Charlottesville, VA, United States of America; 5 Center for Comparative Medicine, University of Virginia, Charlottesville, VA, United States of America; 6 Department of Mechanical Engineering, University of Virginia, Charlottesville, VA, United States of America; 7 Department of Pathology, University of Virginia, Charlottesville, VA, United States of America; The University of Texas Medical Branch, UNITED STATES

## Abstract

DNA-paramagnetic silica bead aggregation in a rotating magnetic field facilitates the quantification of DNA with femtogram sensitivity, but yields no sequence-specific information. Here we provide an original description of aggregation inhibition for the detection of DNA and RNA in a sequence-specific manner following loop-mediated isothermal amplification (LAMP). The fragments generated via LAMP fail to induce chaotrope-mediated bead aggregation; however, due to their ability to passivate the bead surface, they effectively inhibit bead aggregation by longer ‘trigger’ DNA. We demonstrate the utility of aggregation inhibition as a method for the detection of bacterial and viral pathogens with sensitivity that approaches single copies of the target. We successfully use this methodology for the detection of notable food-borne pathogens *Escherichia coli* O157:H7 and *Salmonella enterica*, as well as Rift Valley fever virus, a weaponizable virus of national security concern. We also show the concentration dependence of aggregation inhibition, suggesting the potential for quantification of target nucleic acid in clinical and environmental samples. Lastly, we demonstrate the ability to rapidly detect infectious pathogens by utilizing a cell phone and custom-written application (App), making this novel detection modality fully portable for point-of-care use.

## Introduction

Currently, conventional methods for detection of pathogenic microorganisms (e.g., culture, virus isolation, real-time polymerase chain reaction (qPCR), and immunoassays) are effective, but often time-consuming and dependent on expensive laboratory equipment. The expense of the instrumentation is associated with specifications for the necessary optical properties required for fluorescence detection, which is often laser-induced, or for rapid thermal-cycling [1–2]. To facilitate using nucleic acid amplification tests (NAT) that preclude detection of fluorescence, there is a need for new analytical platforms for amplicon detection. Standard methods for NAT, including qPCR, NASBA, and Loop-mediated isothermal amplification (LAMP), have proven incredibly sensitive and specific for detection of microbial pathogens. However, in order to be portable and cost-effective in a point-of-care (POC) setting, an alternative to these methods is needed for detection of NAT amplicons.

Molecular diagnostic-based detection and characterization of microbial pathogens is of immense utility for emerging diseases, which include multi-drug resistant bacteria and rapidly mutating viruses capable of evading the immune response. Although NAT methods share similar processes for generation of nucleic acid sequence-specific amplicons, the technique of efficient amplicon detection is fundamental to whether this can be done in a portable and cost-effective manner.

In the present investigation we focused on the utilization of paramagnetic silica beads, developed for nucleic acid isolation and image analysis, as a replacement for fluorophores or fluorophore-quenching probes. By eliminating complicated wavelength-specific optical detection and thermal-cycling equipment we abrogate the necessity for expensive and cumbersome instrumentation. We propose that *Product-inhibited Bead Aggregation (PiBA)* represents the potential for rapid and robust detection of pathogen-specific amplicons at a fraction of the cost of conventional methods. We apply this technology to the detection of food-borne pathogens, including *E*. *coli* and *Salmonella*, Rift Valley fever virus (RVFV), a weaponizable RNA virus of national security concern, and human-specific DNA via the thyroid peroxidase (TPOX) gene.

In this report, we demonstrate the sequence-specific detection of both DNA and RNA from human, bacterial, and viral sources with a detection platform that requires only a rudimentary heat source, an inexpensive camera, and a simple computer algorithm. Our description of PiBA as the *inhibition* of chaotropic nucleic acid-induced bead aggregation is the core concept behind this novel detection modality. This method exploits the original use of an existing reagent (paramagnetic beads) to effectively circumvent the need for expensive flourophores or dual-labeled probes. The detection of bead aggregation inhibition involves analysis of an image captured by a camera; inexpensive and portable when compared to the hardware required for laser-induced fluorescence detection and thermal-cycling. DNA and RNA target amplification is driven by loop-mediated isothermal amplification (LAMP). This multi-primer amplification system provides exquisite target specificity and low copy number target sensitivity without the complexities of thermal-cycling and fluorophore detection.

By coupling the PiBA assay to LAMP, we demonstrate that the requirement for thermal-cycling is eliminated, and the assay can effectively be performed on a simple heat block or water bath. The simplicity of both heat control and optical detection makes this technology suitable for resource-limited and rural settings that lack access to important clinical diagnostic facilities.

## Results and Discussion

### Proof of Concept

It has long been established that DNA released from lysed cells binds to silica beads in the presence of a chaotropic salt (e.g., guanidine). The interaction between DNA and the silica surface is thought to be entropically driven, and this phenomenon is the basis for most commercial DNA purification kits used today [3–6]. However, under the same chaotropic conditions in a rotating magnetic field, paramagnetic silica beads are aggregated by DNA, with the extent of aggregation quantitatively linked to the mass of DNA present, a phenomenon referred to as the ‘pinwheel effect’ [7]. The entanglement that leads to DNA-bead aggregation requires a threshold DNA length >10 Kb, and since most DNA released from cells under denaturing conditions is close to full length, direct quantification is possible using this method. This technique can be used directly on crude samples (e.g., whole blood) whereby DNA mass can be quantified and used as a new method for cell counting [8].

Shorter fragments (<3 Kb), however, fail to induce aggregation, being physically too short to entangle the micron-scale beads. While smaller beads (400 nm) have an increased tolerance for shorter DNA, fragments <5 Kb are still problematic. NAT assays generate DNA amplicons <1 Kb in order to facilitate assay speed with specificity; however, it is not possible to use bead aggregation as a detection mode for these short sequences [7].

The inability of short double-stranded nucleic acid fragments to induce entanglement and, subsequently, aggregation, does not mean that these shorter DNA fragments will not bind to the silica bead surface. In fact, there is significant literature that supports this ionic interaction phenomenon [9–10]. For empirical proof, we tested this by evaluating bead aggregation in the presence of short fragments produced by loop-mediated isothermal amplification (LAMP). This amplification approach uses 6–8 primers, specific for a desired DNA (or RNA) target sequence, and generates an abundance of DNA fragments varying in size [11].

**Fig 1:**
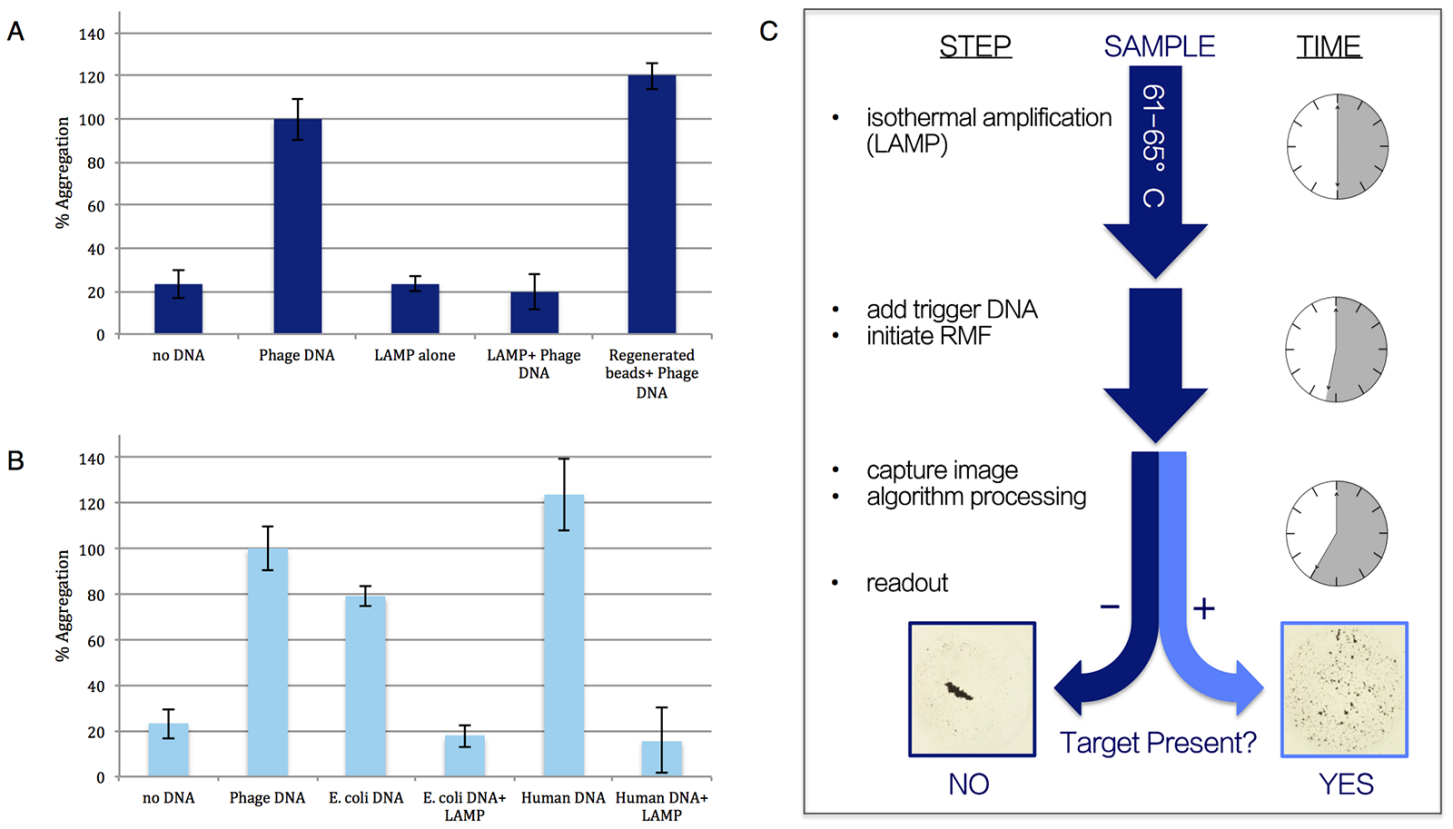
PiBA proof-of-feasibility and mechanism of action. A) Results suggesting LAMP product is too short to induce bead aggregation, but still capable of binding to silica surface of beads and inhibiting aggregation by ‘trigger’ DNA. B) Effect of LAMP product inhibiting aggregation in presence of various types of ‘trigger’ DNA. C) Following LAMP, ‘trigger’ DNA is added to sample and exposed to RMF for 5 minutes. Images are analyzed and a Yes/No read-out is given in a matter of minutes.

Fig 1A shows the result of measuring the impact that an abundance of shorter DNA fragments has on the ability of full length DNA to induce micro-bead aggregation. As expected, the LAMP products (here, *E*. *coli* O157 LAMP product amplified from a starting template of 200 genomic copies) alone failed to induce bead aggregation as a result of inadequate length. More importantly, in the presence of the LAMP products, λ-phage trigger DNA (~48 Kb) fails to induce bead aggregation. Trigger DNA refers to DNA that would induce aggregation of the beads under normal, chaotropic conditions. This supports our theory that small DNA fragments bind avidly to the silica surface and prevent the binding of very long double-stranded DNA fragments that would normally aggregate the beads. When these same beads are “cleansed” of the small double-stranded fragments by elution of bound DNA with TE buffer, exposure anew to λ-phage DNA results in bead aggregation, indicating the integrity of the beads for DNA binding. It is worth noting that λ-phage DNA was set as ‘100% Aggregation’ for these experiments and that the regenerated beads+ phage DNA, as well as the human DNA, both aggregated more than the sample of λ-phage DNA. For this reason, human DNA was used to normalize the data (i.e. set to ‘100% Aggregation’) presented in the remainder of the experiments described in this study.

This data supports our theory that the inhibition of bead aggregation by full length DNA could be an indicator of the presence of an inhibiting concentration of smaller DNA fragments. These fragments effectively compete for a limited number of ionic binding sites on the beads in a non-specific manner with regard to DNA sequence. However, the specificity of LAMP in generating double-stranded DNA fragments is known to be high, owing to multiple primers (no fewer than 6) required for successful product amplification [11], thus it is the inhibition of bead aggregation by trigger DNA that we propose as a new detection modality based on the non-specific binding competition mechanism (S1 Fig). Here, if the smaller fragments of DNA have occupied the binding sites on the beads, full length DNA cannot lash together adjacent beads to induce aggregation. In other words, a standard ‘trigger DNA’ is used to probe the susceptibility of the silica beads to DNA-induced aggregation. This non-specific stoichiometric binding competition is further demonstrated in Fig 1B where DNA from a variety of different sources readily aggregates the beads, but aggregation is consistently inhibited by the presence of LAMP products, supporting the proposed binding competition mechanism. This indicates that (LAMP) fragments, amplified as a result of the presence of a specific nucleic acid target, can be readily detected by inhibition of trigger DNA-induced bead aggregation. Utilizing a cell phone-like camera and a simple algorithm for image analysis, detection through aggregation/image capture/image analysis can yield a Yes/No response in a matter of minutes.

**Fig 2:**
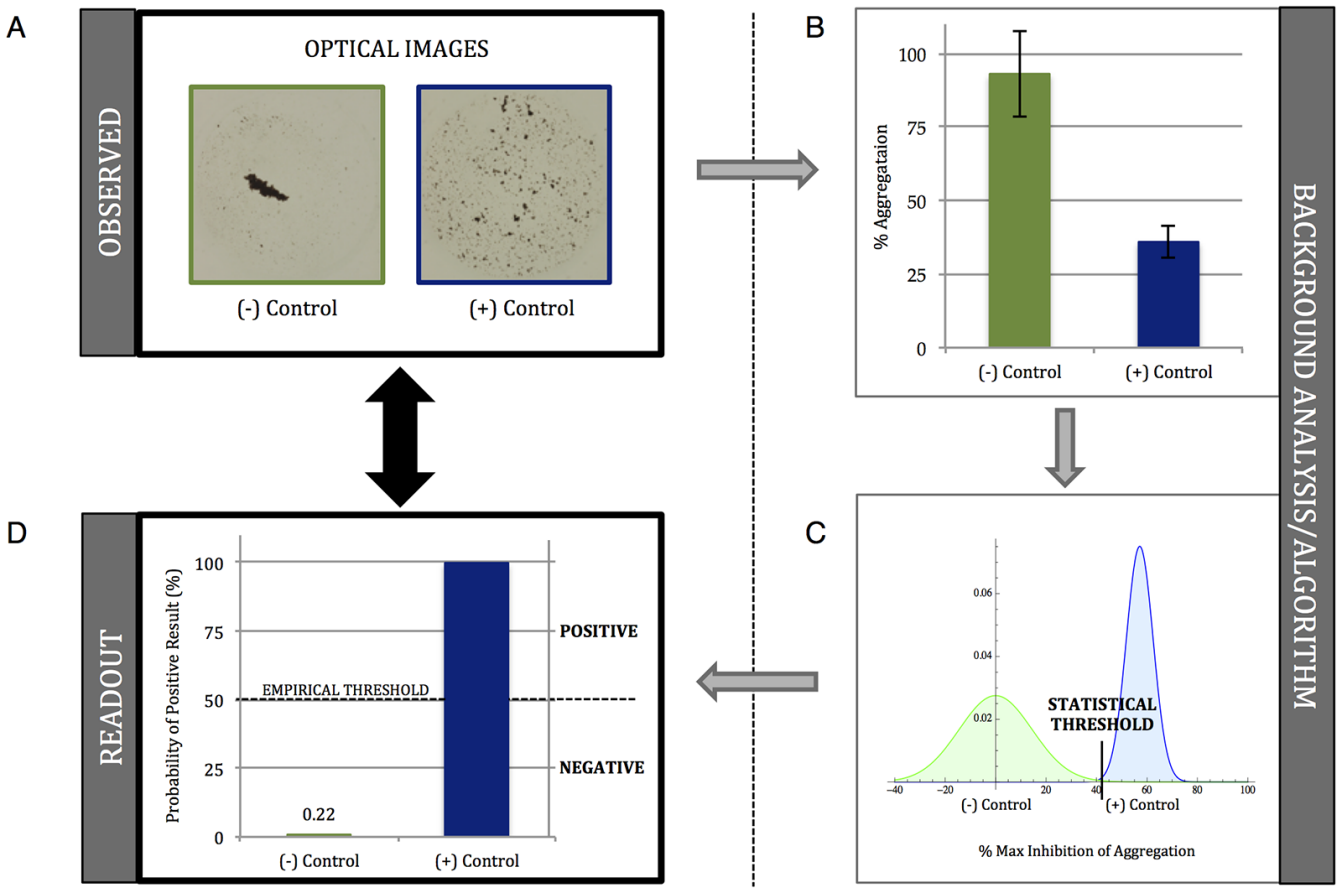
Determining the statistical and empirical (visual) thresholds for genetic analysis via PiBA. A) Optical images of (+/-) Controls. B) Graph of “% Aggregation”, error bars represent standard deviation, n = 3. C) Probability distribution functions (PDF) calculated for (+/-) Controls. Threshold set as [μ-3σ] for (+) Control. X axis shows “% Maximum Inhibition of Aggregation”, (-) Control set as “0%”. D) Graphical representation of PDF. “Probability of Positive Result” represents likelihood of value to cross the calculated statistical threshold. Empirical threshold serves as visual representation of positive/negative results. For all data represented, the empirical threshold is set at 50%. Values above this threshold are considered positive and values below are considered negative.

We sought to evaluate the utility of *Product-inhibited Bead Aggregation* (PiBA) for the detection of DNA and RNA targets specific to a select group of infectious pathogens. In order to accomplish this, aggregation controls had to be established for each target: negative, with no template nucleic acid, and positive, with template for the known target. Optical images are first analyzed, using a Mathematica algorithm, in terms of *“Percent Aggregation”* based on the total number of dark pixels present (Fig 2A and 2B, also S2 Fig). The negative control is then used to normalize the data to “Percent Inhibition of Aggregation” by setting it to 0%. The probability distribution function (PDF) of the positive control is plotted and the statistical threshold is set as 3 times the standard deviation of the mean value. A second Mathematica algorithm then analyzes the PDF of each piece of data (Fig 2C and S3 Fig) and calculates the probability of any given curve crossing over the statistical threshold. Results are presented as “Percent Probability of Positive Result” simply for ease of interpretation so that a Yes/No, qualitative answer is immediately evident when looking at the data. Since this analysis uses both the sample mean and standard deviation in the probability distribution function, error bars are absent in these plots. A visual threshold is determined empirically for ease of result interpretation, and this is shown as 50% for all data presented. Any sample above this threshold is considered positive, while any sample below is considered negative (Fig 2D).

### Food-Borne Pathogen Detection

Pathogen detection in the food industry is important due to the potentially disastrous consequences of failing to detect certain bacteria, and the ensuing public health crisis [12]. The Centers for Disease Control (CDC) estimates that food-borne pathogens account for roughly 128,000 hospitalizations and 3,000 deaths each year in America. *E*. *coli* alone causes approximately 73,000 cases of diarrheal illness in the U.S. each year and further life-threatening complications, including hemolytic uremic syndrome (HUS), occur in about 4% of these cases [13]. Additionally, studies have shown that as few as 10–100 *E*. *coli* cells are enough to cause infection in humans [13–14].

To address this, we wanted to explore the potential utility of this approach for detecting food-borne infectious agents, specifically, *E*. *coli*, *Salmonella*, and *Listeria*. A set of eight unique LAMP primers was used to target sequences in the shiga toxin 2 (stx2) gene of *E*. *coli* O157:H7, strain EDL933 isolated from ground beef associated with the 1982 *E*. *coli* outbreak in Michigan (for primer information, please refer to S1 Table) [15].

**Fig 3:**
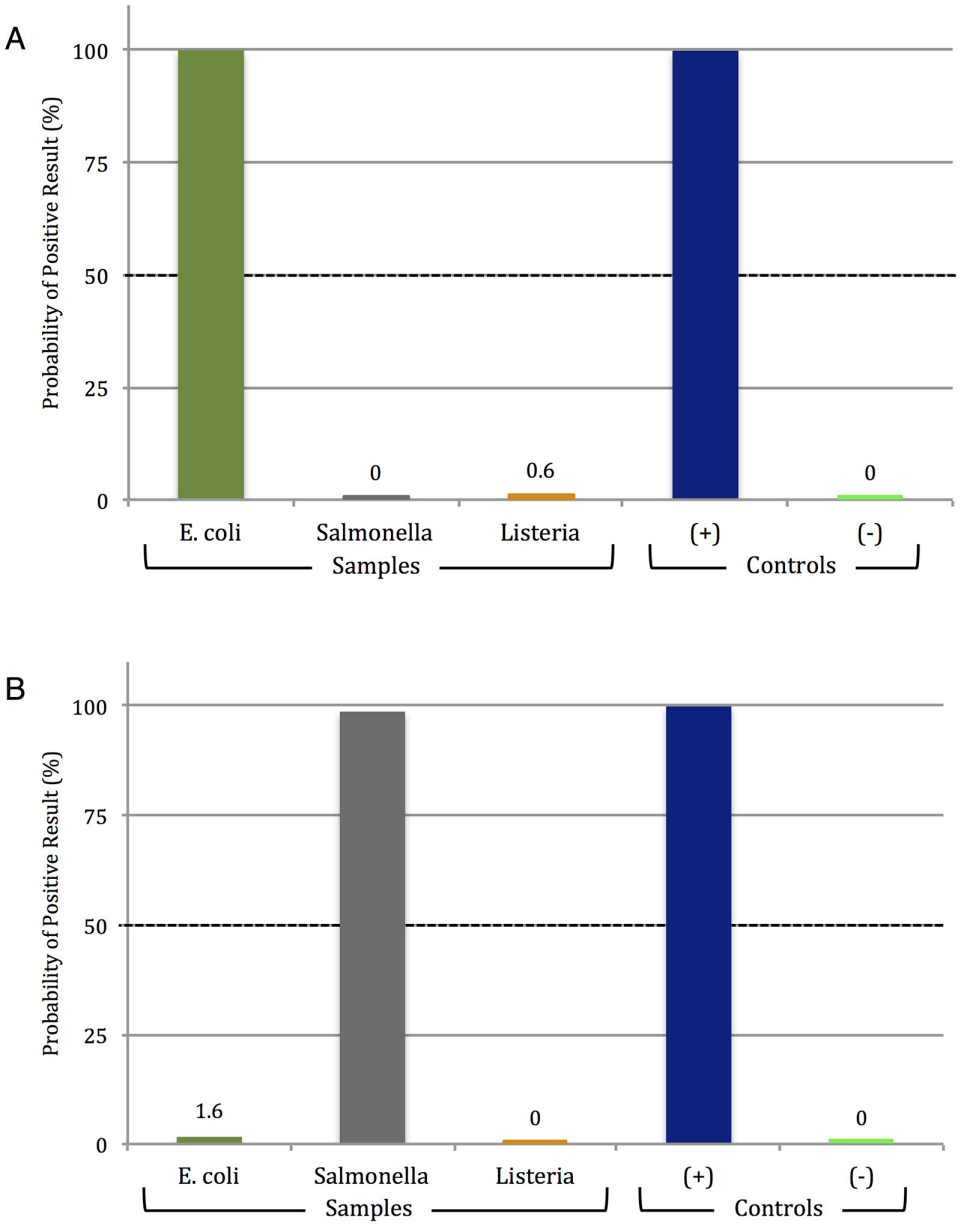
PiBA for specific food-borne pathogen detection. A) PiBA using primers specific to *Escherichia coli*. B) PiBA using primers specific to *Salmonella enterica*. Here, *Listeria monocytogenes* was used as an off-target (-) Control.

Using these, specificity was evaluated, and Fig 3A (corresponding to S4 Fig) shows the clear-cut detection of *E*. *coli* O157:H7; no aggregation inhibition was seen with *Salmonella* or *Listeria* template. The specificity of PiBA was further evaluated by testing the same samples with a primer set specific for target sequences in the Invasion A (invA) gene of *Salmonella* (Fig 3B and S5 Fig). Again, only the desired target organism (*Salmonella*) showed dramatic aggregation inhibition, indicating that PiBA is specific for the bacterial pathogens of interest. In all cases, with both *E*. *coli* and *Salmonella* primers, there is no non-specific amplification of off-target bacterial species, and 200 genomic copies of each target were used for amplification.

**Fig 4:**
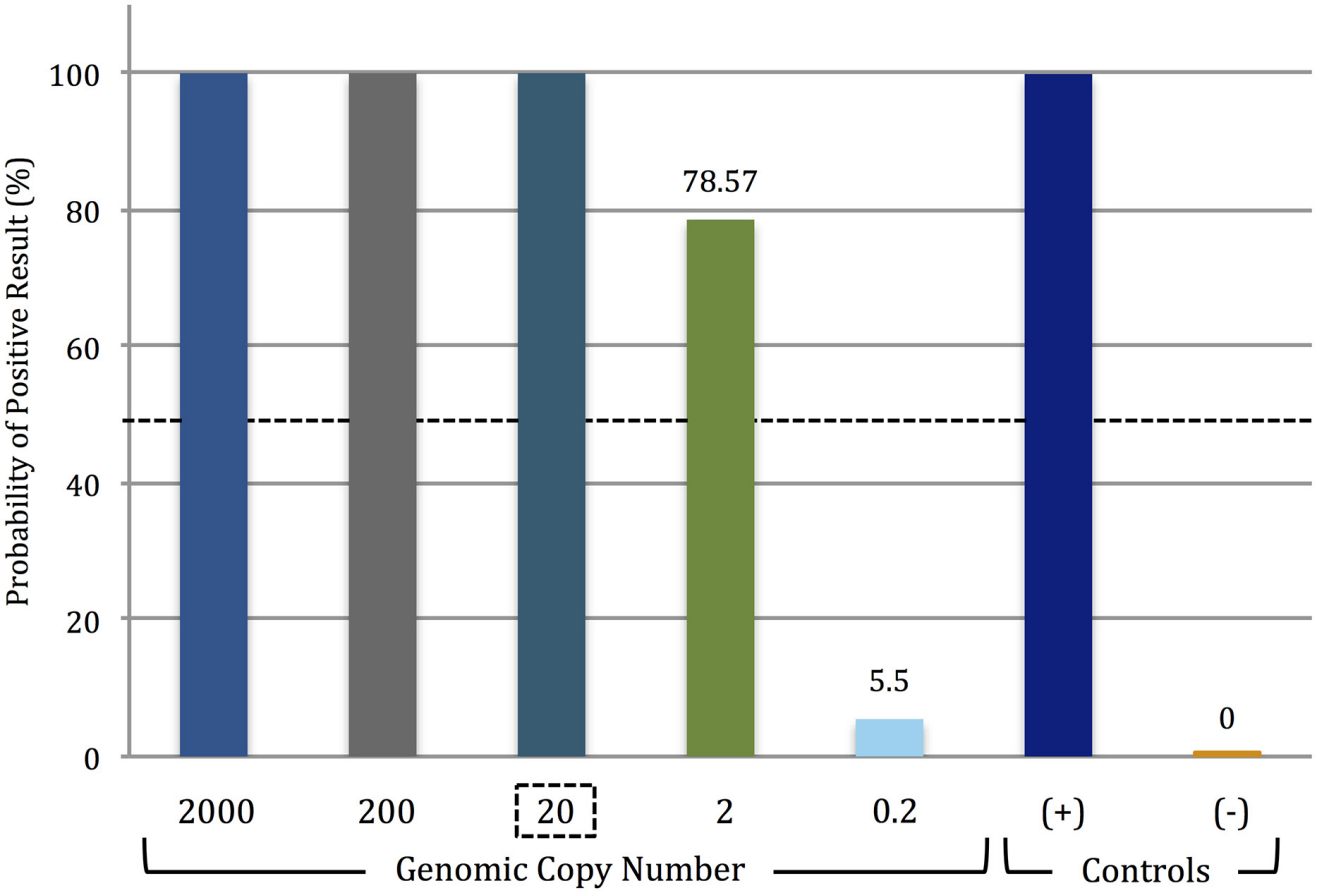
Lower limit of *Escherichia coli* O157:H7 detection via PiBA. Limit of detection was determined to be conservatively 20 copies (2 out of 3 analyses were positive using 2 genomic copies of template DNA). Each starting template amount was tested three times, n = 3. The LOD was determined using *Escherichia coli* O157:H7 strain EDL933 isolated from ground beef.

Having shown (at least with the targets tested) that PiBA had the desired specificity for infectious pathogen detection, the sensitivity of the assay was evaluated using the primer set specific for *E*. *coli* O157:H7 stx2 gene sequences (S1 Table). Solutions prepared through serial dilution provided starting template concentrations that varied from less than 1 to a high of 2000 genomic copies (per amplification reaction). Fig 4 (corresponding to S6 Fig) shows that the aggregation inhibition with template concentrations from 2000 copies down to 20 copies was unequivocal. At a template concentration of 2 copies, significant aggregation inhibition was observed in 2 of 3 analyses. Further dilution to less than 1 copy per reaction (0.2 copies) produced no observable aggregation inhibition. Thus, we conclude that the limit of detection lies around 20 starting copies of genomic DNA. Research has shown that *E*. *coli* contains approximately 4 genomic copies/cell [16]; therefore, our findings are significant for food-borne pathogen detection, as we have demonstrated the ability to detect <10 cells in a sample.

### Detection of *E*. *coli* with Serotype- and Strain-Specificity

Differentiation of various serotypes/strains of the same bacteria has important implications for food-safety and clinical pathology [17]. To address this, *E*. *coli* was used as the model organism. *E*. *coli* O157 affects the food supply and, therefore, has potential impact on patient health. As such, it is critical to have technology capable of detecting bacterial DNA isolated from various types of samples. Enterohemorrhagic (EHEC), Enteroaggregative (EAEC), and Enteropathogenic (EPEC) are three serotypes of *E*. *coli*, and the corresponding strains involved here are O157, O42, and O127, respectively. For the EHEC serotype O157 there are multiple sub-strains, including EDL933, 86–24, and TW14359. The primer set used here is specific to *E*. *coli* O157 and targets the rfbE gene (O-antigen transporter), which is common to all sub-strains (EDL933, 86–24, and TW14359) but absent in O42 and O127. The primer set specific for EAEC O42 targets sequences in the aggR gene (a transcriptional activator of aggregative adherence fimbria) [18], which is absent in the EDL933, 86–24, and TW14359 sub-strains (S1 Table).

**Fig 5:**
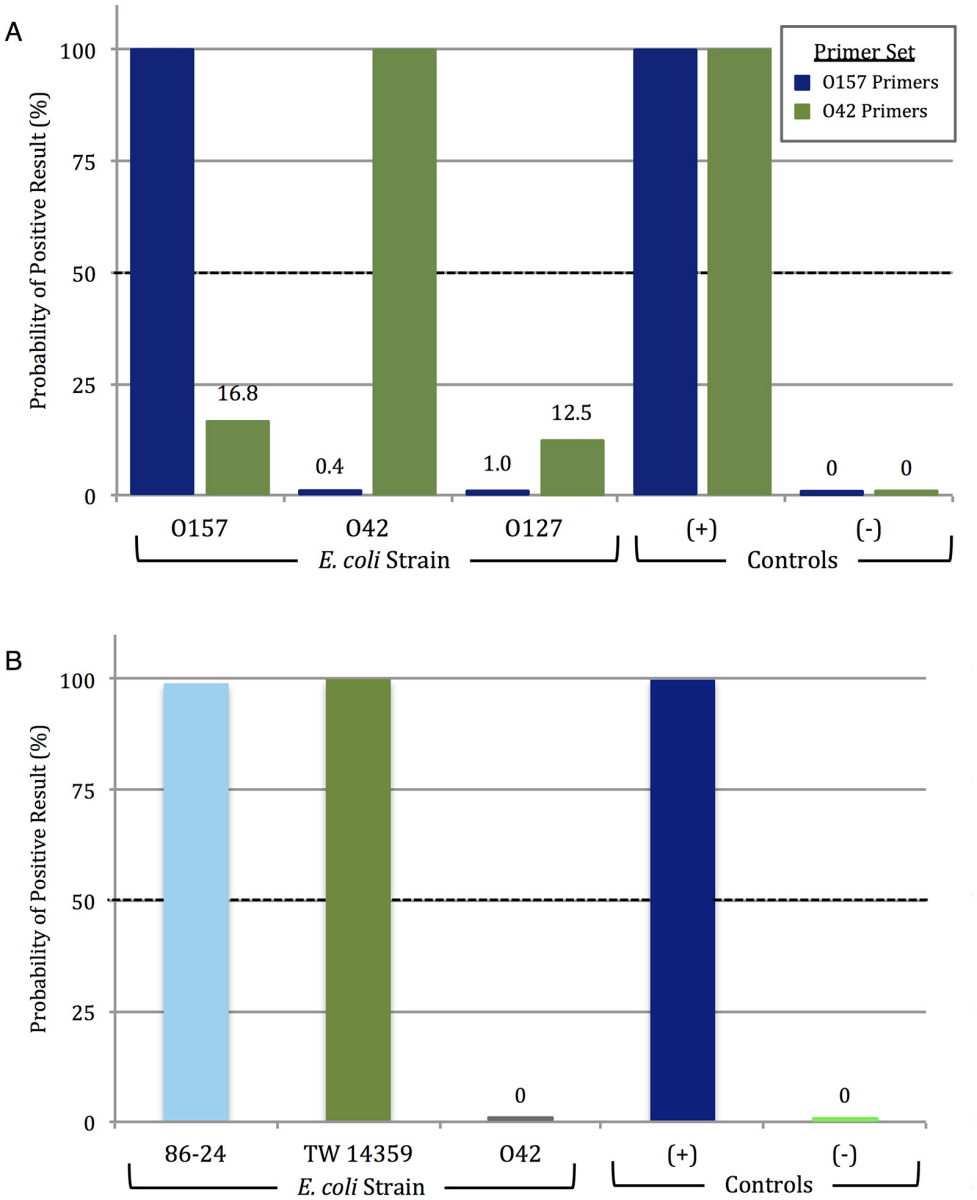
Strain-specific PiBA detection of *E*. *coli*. A) PiBA detection of Enterohemorrhagic *E*. *coli* O157:H7 (EHEC) using primers specific to rfbE gene, shown in blue. Enteroaggregative *E*. *coli* O42 (EAEC) detection using primers specific to aggR gene, shown in green. B) Detection of *E*. *coli* O157 DNA extracted from human stool. 86–24 and TW14359 are O157 strains isolated from outbreaks in 1985 and 2006, respectively. EAEC O42 was used here as an off-target (-) Control.

Fig 5A (corresponding to S7 Fig) shows the results of PiBA detection following amplification of O157, O42, and O127 DNA using the rfbE (O157) and aggR (O42) gene primer sets (200 genomic copies used as starting template for each). Significant aggregation inhibition was only observed with amplified products specific to the target, i.e., O157 primers were largely ineffective with O42 and O127 template DNA. Similar results were obtained with the O42 primers, which were ineffective with the O157 and O127 *E*. *coli* strains. EPEC O127 template DNA was used as an off-target control and, as can be seen in the figure, there was minimal aggregation inhibition observed with either primer set. Again, there is no evidence to suggest non-specific amplification with the O157 primers. However, in experiments with O42 primers there was a slightly higher probability of positive result, though still sufficiently below the empirical threshold. This is attributed to primer design, and continued optimization of both primer sequence and amplification temperature in future studies will likely decrease this.

Having successfully demonstrated detection of *E*. *coli* O157 isolated from ground beef, we sought to further validate the LAMP-PiBA assay using the same starting template amount with human stool samples associated with other outbreaks. O157 strain TW14359 was responsible for the spinach-associated outbreak in 2006, while strain 86–24 was associated with an outbreak in 1985 traced back to contaminated beef [13]. Results in Fig 5B (corresponding to S8 Fig) show that both TW14359 and 86–24 exhibit aggregation inhibition, correlating with a high probability of a positive result. A complete lack of aggregation inhibition with *E*. *coli* O42 indicates minimal (or no) non-specific amplification. These results support the potential for future use of PiBA with various starting sample types (food, human stool).

### Human-Specific DNA Detection

**Fig 6:**
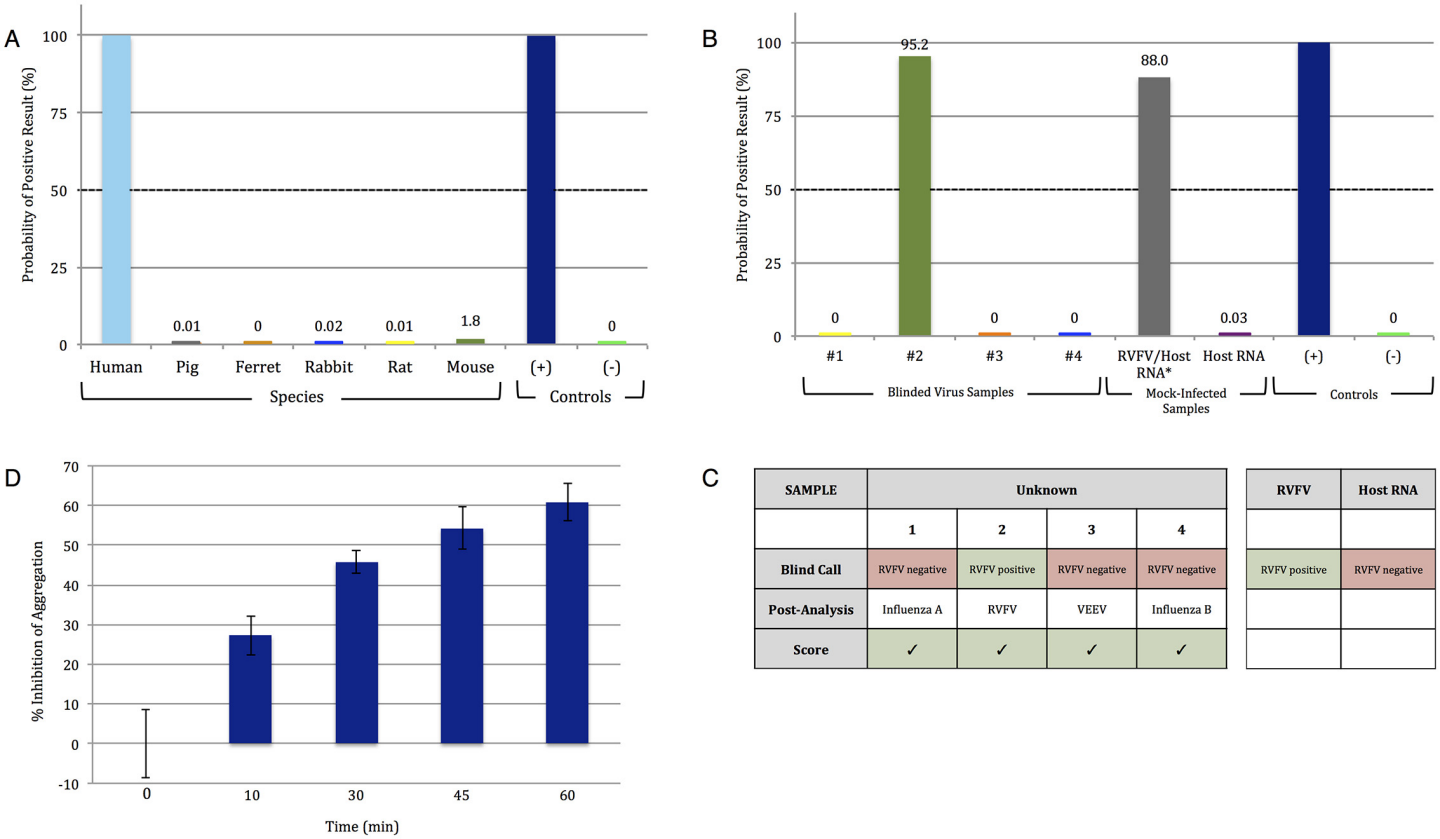
Detecting the presence of DNA specific to the human thyroid peroxidase (TPOX) gene and Rift Valley fever virus (RVFV) RNA. A) PiBA for detection of human-specific DNA from whole blood. Primers were designed to target the TPOX gene. B) RVFV detection from infected (viral RNA in a background of host RNA) samples. #1–4 represent blinded viral samples. C) Results of blinded analysis of samples. D) Results suggesting that “% Inhibition of Aggregation” is concentration dependent and varies with time of amplification.

To demonstrate the full bandwidth of the LAMP-PiBA assay, we focused on human genomic DNA pertinent to a forensic application. For forensic DNA analysis, where a number of genetic loci are probed for tetra- and penta-nucleotide repeats, it is critical to define that casework samples have DNA of human origin. The thyroid peroxidase (TPOX) gene is a commonly probed locus that is human-specific. To test PiBA in this capacity, LAMP primers were specifically designed to target the TPOX gene, and used for amplification of DNA from multiple species with a starting template concentration of 1ng/μL (S1 Table). As shown in Fig 6A (corresponding to S9 Fig), only human DNA elicited a response that was consistent with the presence of the TPOX sequence.

### Virus Detection

Having shown success of the LAMP-PiBA assay for detection of bacterial DNA, we were curious as to its effectiveness with RNA and virus targets. The capability of PiBA for detection of an RNA virus is demonstrated here for RVFV-MP12, a mosquito-borne pathogen, which along with other viruses such as Ebola, is known to cause viral hemorrhagic fever (VHF). In 1999, the CDC classified all viruses capable of producing VHF as Category A Bioweapon Agents, due in part to the lack of vaccines available to the general public [19]. RVFV outbreaks occur routinely in rural parts of Africa and the ability to have a PiBA-based POC platform would be extremely beneficial for field diagnostics.

In order to detect RVFV-MP12, LAMP primers specific for the L Polymerase gene (L pol) were utilized (S1 Table). Four blinded viral samples (purified RNA), potentially containing RVFV MP12, were obtained and tested using L pol primers. Fig 6B (corresponding to S10 Fig) shows that, of the four samples, only sample #2 was positive for RVFV-MP12.

The scoring of the trials with blinded samples is given in Fig 6C. With samples #1, 3, and 4 containing RNA template from Influenza A, Influenza B, and Venezuelan Equine Encephalitis Virus (VEEV TC83), respectively, the reverse transcription (RT)-LAMP-PiBA assay proved effective in defining the only sample with RVFV-MP12 (#2). This was confirmed by conventional qRT-PCR (S11 Fig).

Also positive was the RVFV-MP12 infected sample which consisted of RVFV RNA in a background of host RNA at a ratio of ~1: 10,000. This is significant as it suggests that RT-LAMP-PiBA may have potential for use with samples that have not undergone upstream enrichment procedures. Additionally, it proves the feasibility to perform the PiBA assay following RT-LAMP, as well as LAMP. By demonstrating proof of feasibility with various bacterial and viral pathogens in the present study, we believe our technique could reasonably be extended to apply to any target pathogen for which specific primers can be designed.

Fig 6D shows some final results that are noteworthy. With PCR, there is an exponential increase in the number of amplified product copies with each cycle, and this can be observed in real-time with fluorescence detection. Since LAMP is time-based, not cycle-based, it stands to reason that there should be an increase in the mass of amplified product with time. However, despite the sensitivity of PiBA (see Fig 4) it was not obvious that amplicon production would be observable in a similar manner using nothing more than bead aggregation as a read-out. Given the time-course results for RVFV-MP12 shown in Fig 6D, it appears that it may be possible to quantify amplicon production based on the extent of bead aggregation inhibition. Future studies will investigate the effect of bead size and concentration on the sensitivity and the dynamic range of the PiBA assay, while our preliminary studies suggest the dynamic range of aggregation inhibition lies between 20–2000 genomic copies.

As mentioned above, PiBA can be utilized for human-specific DNA discrimination, and if the quantitative potential is fully realized with this assay, it could prove powerful for use upstream of STR analysis by providing an answer in terms of human specificity and amount of DNA present.

### Conclusions

Fluorescence dominated as a sensitive detection modality for nucleic acid testing, and obviously played a key role in the sequencing of the human genome [20–21]. While powerful, it is tethered to the use of specialized reagents that must be purchased commercially, and are often expensive. In addition, hardware demands are high when lasers are used for excitation and sophisticated optical systems are required for detection. Traditional LAMP can be interpreted by comparing the turbidity; however, this can lead to subjectivity and misinterpretation on the part of the reader if they are untrained. For point-of-care devices, it is advantageous to have test results that are presented in a simple, Yes/No way to avoid misinterpretation. It is for these reasons that there is intense interest in label-free detection technologies. If inexpensive reagents and simplified optical components are involved, a more portable and cost-effective instrument becomes possible. With LAMP-PiBA, reagent requirements include only fluor-free primers for amplification, unlabeled commercially-available magnetic beads, and guanidine. Likewise, detection with PiBA consists of simple image capture and analysis with an inexpensive cell phone-like camera. In this respect, PiBA as a label-free detection technology is the antithesis of fluorescence detection.

In this report, we make a case for the broad range utility of LAMP-PiBA, showing its effectiveness for sequence-specific DNA and RNA detection, as well as applicability to bacteria, virus, and human sequences. On the amplification front, specificity is driven by the 6–8 primers needed for LAMP, where incomplete hybridization of any one primer results in no amplification [22]. Comparing the per run reagent costs for LAMP with PCR, the primer cost differential is negligible at ~7¢ and 1¢, respectively, while the fluorescence reagents for qPCR (SYBR green or Taqman probes) total ~$1; cost of beads for PiBA is a fraction of a cent. Consequently, the overall cost differential is ~10-fold.

**Fig 7:**
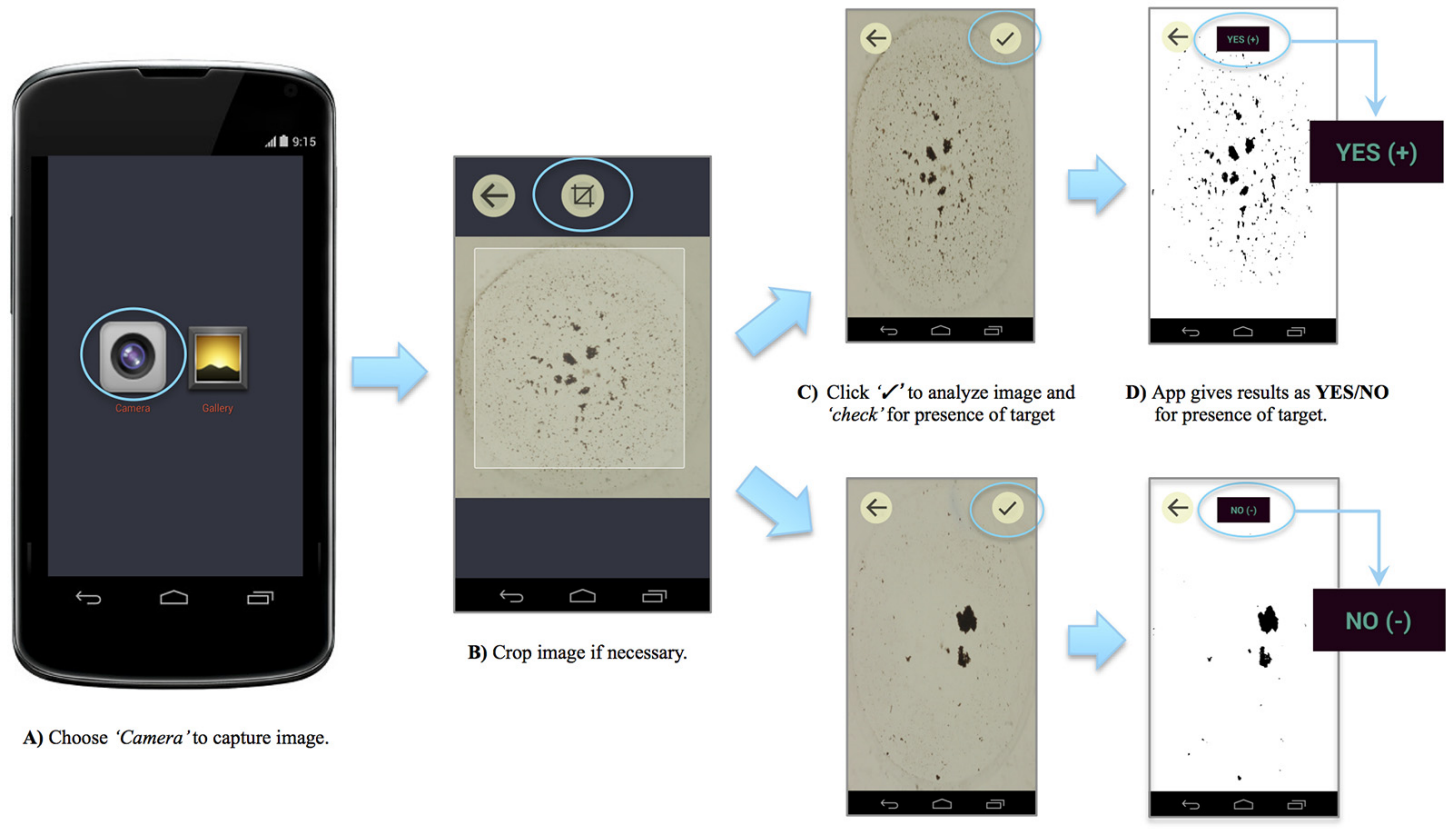
Detection of *Salmonella* using custom Android app. A) Choose camera setting to capture an image of the microwell. B) Crop the image if necessary. C) The image is analyzed using a custom-written algorithm to calculate “Percent Aggregation”. D) Results are given as a simple YES/NO output for ease of interpretation.

While cost reduction is not without value, the true paradigm shift with PiBA is in the hardware. The ‘pinwheel effect’ was discovered using the rotating magnetic field generated by a laboratory stir plate. This is currently being miniaturized as an electromagnetic array that interrogates 5 mm microwells in a small (2 cm^2^) microchip. The excitation source (lamp or laser) and photo-detection system (optics, photodiode, CCD) required for fluorescence detection are circumvented in PiBA by a camera (minimum 3 MP) for image capture with no need for elaborate back-lighting. Together, these represent a significant simplification in the hardware for detection. The image analysis (an algorithm in *Mathematica*) is simple and can be converted into a cell phone application (app). In fact, Fig 7 shows the processing of images from *Salmonella* samples, and the resultant Yes/No call is given as a screen shot of the Android phone. These characteristics make PiBA a potentially powerful tool in resource-limited settings where infectious disease diagnostics are of critical importance. Cited as an invaluable criteria for point-of-care testing, simple *‘Yes/No detection’* is required in order to avoid misinterpretation of results by untrained users [23].

Based on these preliminary results and the successful utilization of PiBA with a cell phone camera and app (Fig 7), we believe this technology represents the potential for a fully-automated, inexpensive, and portable device suitable for resource-limited settings. Integration onto a rotationally-driven microdevice (RDM), costing <$1 to fabricate, would allow for multiplexed results in 10s of minutes. Such a device could be driven by the equivalent of a Sony Discman, relying solely on battery power- significant because of the departure from conventional electricity-dependent devices. The potential of PiBA to be integrated into such a device stems from the limited equipment requirements: basic heat source, cell phone camera, and custom app.

## Materials and Methods

### LAMP/RT-LAMP Protocol

All LAMP primer sequences and amplification temperatures can be found in S1 Table. Primers were purchased from Eurofins MWG Operon. Reactions were carried out using a LoopAmp DNA Amplification Kit and a LoopAmp RNA Amplification Kit (Eiken Chemical Co., Ltd. Tokyo, Japan). For all reactions the following amounts of primers were used: 5 pmol each of F3 and B3, 20 pmol each LF and LB, and 40 pmol each of FIP and BIP. Positive and negative controls were included in each run. For primers designed in-house, LAMP PrimerExplorer V4 software was used (http://primerexplorer.jp/elamp4.0.0/index.html; Eiken Chemical Co., Ltd. Tokyo, Japan).

### DNA/RNA Extraction

#### Human and Animal

DNA was extracted from human and animal blood for TPOX amplification using a QIAamp DNA mini kit (Qiagen, Netherlands) according to manufacturer protocol. Animal samples were provided by the University of Virginia Center for Comparative Medicine in accordance with the University of Virginia Institutional Animal Care and Use Committee (ACUC). Blood collection was specifically approved by the UVA ACUC for diagnostic and research purposes under the animal protocol used in this study. Blood was collected from the following animals: pig, ferret, rabbit, rat, and mouse. Human samples were de-identified, prior to the authors receiving them, and scheduled for discard by the University of Virginia Medical Laboratories. All samples were stored at -20°C until use.

#### Bacteria

*E*. *coli* cultures were grown overnight in LB media (Fisher Scientific) with antibiotics to an O.D.600 of approximately 1.5. Strains EDL933, 86–24, TW14359, and O42 were grown with streptomycin (Sigma-Aldrich), final concentration 100μg/mL. Strain O127 (E2348/69) was grown in nalidixic acid (Sigma-Aldrich), final concentration 100μg/mL. Strain 86–24 was isolated from a patient in 1986 [24], strain O42 was isolated from an infected patient in 1983 [25], strain O127 was isolated from a case of infantile diarrhea in 1969 [26], strain TW14359 was isolated from a spinach outbreak in 2006 [27], and strain EDL933 was isolated from raw hamburger (ground beef) implicated with outbreaks in 1982 [28]. These samples were generously provided by Dr. Kendall at the University of Virginia. DNA was extracted using 1mL of culture and a GenElute Bacterial Genomic DNA Kit (Sigma Aldrich, St. Louis, MO). Genomic DNA was eluted with water and allowed to sit for five minutes prior to centrifugation. All samples were stored at -20°C until use.

#### Virus

Rift Valley fever virus (RVFV) strain MP12, a recombinant vaccine strain (arMP12), was generated using an RVFV reverse-genetics system and then passaged in Vero cells. The vaccine strain was originally derived from the RVFV ZH548 strain that had been isolated in 1977 from a patient with uncomplicated RVFV [29]. These samples were generously provided by the University of Texas Medical Branch, Galveston, TX. The Venezuelan Equine Encephalitis Virus (VEEV) strain TC83 was obtained from BEI Resources (NR63). VEEV TC83 was propagated by infecting Vero cells at 80–90% confluence at an MOI of 0.1 in supplemented media. Influenza A/California/04/09 and Influenza B/Taiwan/2/62 strains of Influenza viruses were obtained from BEI Resources. Influenza viruses were propagated by infecting MDCK cells at 80–90% confluence at an MOI of 0.1 in Influenza Growth Media (DMEM supplemented with 1% bovine serum albumin, 1% non-essential amino acids, 1% L-glutamine, and 1% penicillin/streptomycin). TPCK-treated trypsin from bovine pancreas (Sigma-Aldrich) was added to the Influenza Growth Media at a final concentration of 200 ng/mL. Viral titers were determined by plaque assays as previously described [30–32].

Viral RNA was isolated from cell-free viral supernatants using Trizol LS according to manufacturer’s recommendations (Life Technologies, Inc.) and DNase treated. RNA quantification was performed using the Quant-iT RiboGreen RNA assay (Invitrogen) using the DTX 880 Multimode Detector plate reader (Beckman Coulter). The concentration of the sample was determined using the Multimode Analysis software (Version 3.3.09). All samples (RVFV and the four blinded RNA samples) were then diluted to 5E+07 genomic copies/5 μL. The final concentration (in g/μL) for 5E+07 genomic copies per 5μL was determined by multiplying the mass of the genome (1.6E-17 for RVFV; L, M, and S segment genome sizes are added together) by the genomic copies wanted (5E+07), then dividing the number by 5μL. The final volume for 5E+07 per 5μL was determined by multiplying the stock sample concentration by the stock sample volume, then dividing the number by the concentration wanted (for RVFV, 0.16 ng/μL for 5E+07 genomic copies per 5μL). Total RNA was extracted from mock-infected (media containing no virus) and MP12 infected Vero cells using RNeasy Mini Kit (Qiagen). RNA quantification was performed using the NanoDrop 2000 Spectrophotometer (Thermo Scientific).

### Quantitative real-time PCR

For RVFV and VEEV samples, qRT-PCR with viral specific primers and TaqMan fluorogenic probes was performed using RNA UltraSense One-Step Quantitative RT-PCR System (Life Technologies) as previously described. For Influenza A and Influenza B samples, qRT-PCR with viral specific primers (S11 Fig) was performed using the SYBR green real-time PCR System (Life Technologies).

### Reagent preparation

30μL of stock Magnesil beads (purchased from Promega, Madison, WI) were washed three times with guanidine hydrochloride (GdnHCl) solution (8M, 1X TE, adjusted to pH 6.1 with 100 mM MES) and resuspended in a total volume of 1000μL GdnHCl solution.

### PiBA

The following was added to each 20μL well: 13μL of GdnHCl solution, 4μL stock Magnesil beads, 2μL LAMP sample, 0.5μL pre-purified human genomic DNA (‘trigger’ DNA, 1.0 ng/μL, purchased from Promega). Mixture was exposed to a rotating magnetic field (RMF) at 2200 rpm for 5 min and vortexer was used at ~500 rpm to agitate the samples during assay. Images of microwells were captured using a Canon Rebel EOS Rebel T1i, 15.1-megapixel camera. Image files were processed using an isodata algorithm written in Mathematica software.

## Supporting Information

S1 Fig Schematic of PiBA mechanism. LAMP product is added to a sample of magnetic beads. If the amplification was successful, the presence of short fragments of the target sequence inhibits aggregation by trigger DNA.
(TIFF)

S2 Fig Sample output from Mathematica algorithm. Images of each microwell are analyzed following the PiBA assay. “Percent Aggregation” is calculated based on the number of dark pixels present in each image.
(TIFF)

S3 Fig Sample probability distribution functions. PDFs are plotted for each piece of data (here, *E*. *coli* primer specificity) and the statistical threshold is calculated as 3 times the standard deviation of the mean of the positive control. A Mathematica algorithm is used to calculate the probability of any given curve crossing the statistical threshold.
(TIFF)

S4 Fig Results of *E*. *coli* primers. Data from Fig 3A presented as % Inhibition of Aggregation.
(TIFF)

S5 Fig Results of *Salmonella* primers. Data from Fig 3B presented as % Inhibition of Aggregation.
(TIFF)

S6 Fig Results of Limit of Detection experiments. Data from Fig 4 presented as % Inhibition of Aggregation.
(TIFF)

S7 Fig Results of *E*. *coli* strain specificity experiments. Data from Fig 5A presented as % Inhibition of Aggregation.
(TIFF)

S8 Fig Results of *E*. *coli* experiments extracted from human stool. Data from Fig 5B presented as % Inhibition of Aggregation.
(TIFF)

S9 Fig Results of Human-specific DNA experiments. Data from Fig 6A presented as % Inhibition of Aggregation.
(TIFF)

S10 Fig Results of RVFV experiments. Data from Fig 6B presented as % Inhibition of Aggregation.
(TIFF)

S11 Fig Results from RVFV qRT-PCR. A) Table of results from RVFV qRT-PCR. B) Graph of genomic copies per reaction, confirming blinded viral sample results. C) Primer sequences used.
(TIFF)

S1 Table LAMP primer sequences and amplification temperatures for all targets. A) *E*. *coli* stx2, 65°C [33]. B) *Salmonella* invA, 63°C [34]. C) *E*. *coli* O157 rfbE, 65°C [22]. D) *E*. *coli* O42 aggR, 65°C. E) Human-specific TPOX, 0062°C. F) RVFV L polymerase, 61°C [35].
(TIFF)

## References

[1] Leland D, Ginocchio C (2007) Role of cell culture for virus detection in the age of technology. Clin Microbiol Rev 20(1):49–78. 17223623 10.1128/CMR.00002-06 PMC1797634

[2] Kiilerich-Pedersen K, Daprà J, Cherré S, Rozlosnik N (2013) High sensitivity point-of-care device for direct virus diagnostics. Biosens Bioelectron 49:374–379. 10.1016/j.bios.2013.05.046 23800609

[3] Bienvenue J, Duncalf N, Marchiarullo D, Ferrance J, Landers JP (2006) Microchip-based cell lysis and DNA extraction from sperm cells for application to forensic analysis. J Forensic Sci 51:266–273. 16566759 10.1111/j.1556-4029.2006.00054.x

[4] Breadmore M, Wolfe K, Arcibal I, Leung W, Dickson D, Giordano B, et al (2003) Microchip-based purification of DNA from biological samples. Anal Chem 75:1880–1886. 12713046 10.1021/ac0204855

[5] Hagan K, Bienvenue J, Muskaluk C, Landers JP (2008) Microchip-based solid-phase purification of RNA from biological samples. Anal Chem 80:8453–8460. 10.1021/ac8011945 18855414

[6] Tian H, Huhmer A, Landers JP (2000) Evaluation of silica resins for direct and efficient extraction of DNA from complex biological matrices in a miniaturized format. Anal Biochem 283:175–191. 10906238 10.1006/abio.2000.4577

[7] Leslie D, Li J, Strachan B, Begley M, Finkler D, Bazydlo L, et al (2012) New detection modality for label-free quantification of DNA in biological samples via superparamagnetic bead aggregation. J Am Chem Soc 134:5689–5696. 10.1021/ja300839n 22423674 PMC3339050

[8] Li J, Liu Q, Xiao L, Haverstick D, Dewald A, Columbus L, et al (2013) Label-free method for cell counting in crude biological samples via paramagnetic bead aggregation. Anal Chem 85(23):11233–11239. 10.1021/ac401402h 24187938

[9] Boom R, Sol C, Salimans M, Jansen C, Werthein-van Dillen P, van der Noordaa J (1990) Rapid and simple method for purification of nucleic acids. J Clin Microbiol 28(3):495–503. 1691208 10.1128/jcm.28.3.495-503.1990 PMC269651

[10] Vogelstein B, Gillespie D (1979) Preparative and analytical purification of DNA from agarose. Proc Natl Acad Sci 76(2):615–619. 284385 10.1073/pnas.76.2.615 PMC382999

[11] Notomi T, Okayama H, Masubuchi H, Yonekawa T, Watanabe K, Amino N, et al (2000) Loop-mediated isothermal amplification of DNA. Nucleic Acids Res 28(12):e63 10871386 10.1093/nar/28.12.e63 PMC102748

[12] Lazcka O, Del Campo F, Muñoz F (2007) Pathogen detection: A perspective of traditional methods and biosensors. Biosens Bioelectron 22:1205–1217. 16934970 10.1016/j.bios.2006.06.036

[13] Mohawk K, Melton-Celsa A, Zangari T, Carroll E, O’Brien A (2010) Pathogenesis of *Escherichia coli* O157:H7 strain 86–24 following oral infection of BALB/c mice with an intact commensal flora. Microb Pathogenesis 48:131–142. 10.1016/j.micpath.2010.01.003 20096770 PMC2834854

[14] Yao Z, Wei G, Wang H, Wu L, Wu J, Xu J (2013) Survival of *Escherichia coli* O157:H7 in soils from vegetable fields with different cultivation patterns. App Environ Microb 79(5):1755–1756. 10.1128/AEM.03605-12 PMC3591966 23291546

[15] Riley L, Remis R, Helgerson S, McGee H, Wells J, Davis B, et al (1983) Hemorrhagic colitis associated with a rare *Escherichia coli* serotype. New Engl J Med 308(12):681–685. 6338386 10.1056/NEJM198303243081203

[16] Luisi PL, Stano P (2010) The minimal cell: the biophysics of cell compartment and the origin of cell functionality A biophysical chemists thoughts about the lower limit of cell sizes (Springer), pp 65–71.

[17] Paton J, Paton A (1998) Pathogenesis and diagnosis of shiga toxin-producing *Escherichia coli* infections. Clin Microbiol Rev 11(3):450–479. 9665978 10.1128/cmr.11.3.450 PMC88891

[18] Nataro J, Yikang D, Yingkang D, Walker K (1994) AggR, a transcriptional activator of aggregative adherence fimbria I expression in enteroaggregative *Escherichia coli* . J Bacteriol 176(15):4691–4699. 7913930 10.1128/jb.176.15.4691-4699.1994 PMC196291

[19] Narayanan A, Bailey C, Kashanchi F, Kehn-Hall K (2011) Developments in antivirals against influenza, smallpox and hemorrhagic fever viruses. Expert Opin Investig Drugs 20(2):239–254. 10.1517/13543784.2011.547852 21235430 PMC9476113

[20] Schena M, Shalon D, Heller R, Chai A, Brown P, Davis R (1996) Parallel human genome analysis: Microarray-based expression monitoring of 1000 genes. Proc Natl Acad Sci 93:10614–10619. 8855227 10.1073/pnas.93.20.10614 PMC38202

[21] Lander E, Linton L, Birren B, Nusbaum C, Zody M, Baldwin J, et al (2001) Initial sequencing and analysis of the human genome. Nature 409:860–921. 11237011 10.1038/35057062

[22] Zhao X, Li Y, Wang L, You L, Xu Z, Li L, et al (2010) Development and application of a loop-mediated isothermal amplification method on rapid detection of *Escherichia coli* O157 strains from food samples. Mol Biol Rep 37:2183–2188. 10.1007/s11033-009-9700-6 19685165

[23] Yager P, Edwards T, Fu E, Helton K, Nelson K, Tam M, et al (2006) Microfluidic diagnostic technologies for global public health. Nature 442(27):412–418. 16871209 10.1038/nature05064

[24] Griffin P, Ostroff S, Tauxe R, Greene K, Wells J, Lewis J, et al (1988) Illnesses associated with *Escherichia coli* O157:H7 infections: a broad clinical spectrum. Ann Intern Med 109(9):705–712. 3056169 10.7326/0003-4819-109-9-705

[25] Chaudri R, Sebaihia M, Hobman J, Webber M, Leyton D, Goldberg M, et al (2010) Complete genome sequence and comparative metabolic profiling of the prototypical enteroaggregative *Escherichia coli* strain O42. PLoS ONE 5(1):e8801 10.1371/journal.pone.0008801 20098708 PMC2808357

[26] Wu SX, Peng RQ (1991) Studies on adherence and outer membrane protein of enteropathogenic *Escherichia coli* O127:H6 and their related plasmids. Acta Paediatr Scand 80(11):1019–1024. 1750334 10.1111/j.1651-2227.1991.tb11777.x

[27] California Food Emergency Response Team (2007). Investigation of an *Escherichia coli* O157:H7 outbreak associated with Dole pre-packaged spinach. California Department of Health Services, Sacramento, CA.

[28] Wells J, Davis B, Wachsmuth I, Riley L, Remis R, Sokolow R, et al (1983) Laboratory investigation of hemorrhagic colitis outbreaks associated with a rare *Escherichia coli* serotype. J Clin Microbiol 18(3):512–520. 6355145 10.1128/jcm.18.3.512-520.1983 PMC270845

[29] Ikegami T, Makino S. (2009) Rift Valley fever vaccines. Vaccine 27:D69–D72. 10.1016/j.vaccine.2009.07.046 19837291 PMC2764559

[30] Szretter K, Balish A, Katz J. (2005) Curr Protoc Microbiol (John Wiley & Sons, Inc.). Available: http://onlinelibrary.wiley.com/doi/10.1002/0471729256.mc15g01s3/abstract.

[31] Shafagati N, Narayanan A, Baer A, Fite K, Pinkham C, Bailey C, et al (2013) The use of NanoTrap particles as a sample enrichment method to enhance the detection of Rift Valley fever virus. PLoS Negl Trop Dis 7:e2296 10.1371/journal.pntd.0002296 23861988 PMC3701711

[32] Lundberg L, Pinkham C, Baer A, Amaya M, Narayanan A, Wagstaff K, et al (2013) Nuclear import and export inhibitors alter capsid protein distribution in mammalian cells and reduce Venezuelan Equine Encephalitis Virus replication. Antiviral Res 100:662–672. 10.1016/j.antiviral.2013.10.004 24161512

[33] Wang F, Jiang L, Ge B (2011) Loop-mediated isothermal amplification assays for detecting shiga toxin-producing *Escherichia coli* in ground beef and human stools. J Clin Microbiol 50(1):91–97. 10.1128/JCM.05612-11 22031701 PMC3256711

[34] Chen S, Wang F, Beaulieu J, Stein R, Ge B (2011) Rapid detection of viable Salmonellae in produce by coupling propidium monoazide with loop-mediated isothermal amplification. Appl Environ Microbiol 77(12):4008–4016. 10.1128/AEM.00354-11 21498750 PMC3131628

[35] LeRoux C, Kubo T, Grobbelaar A, Jansen van Vuren P, Weyer J, Nel L, et al (2009) Development and evaluation of a real-time reverse transcription-loop-mediated isothermal amplification assay for rapid detection of Rift Valley fever virus in clinical specimens. J Clin Microbiol 47(3):645–651. 10.1128/JCM.01412-08 19109471 PMC2650915

