# Supplementary figures and images for: Optical Imaging of Paramagnetic Bead-DNA Aggregation Inhibition Allows for Low Copy Number Detection of Infectious Pathogens
Source: PLoS One. 2015 Jun 11;10(6):e0129830. doi: 10.1371/journal.pone.0129830 (PMC4466016; doi:10.1371/journal.pone.0129830)

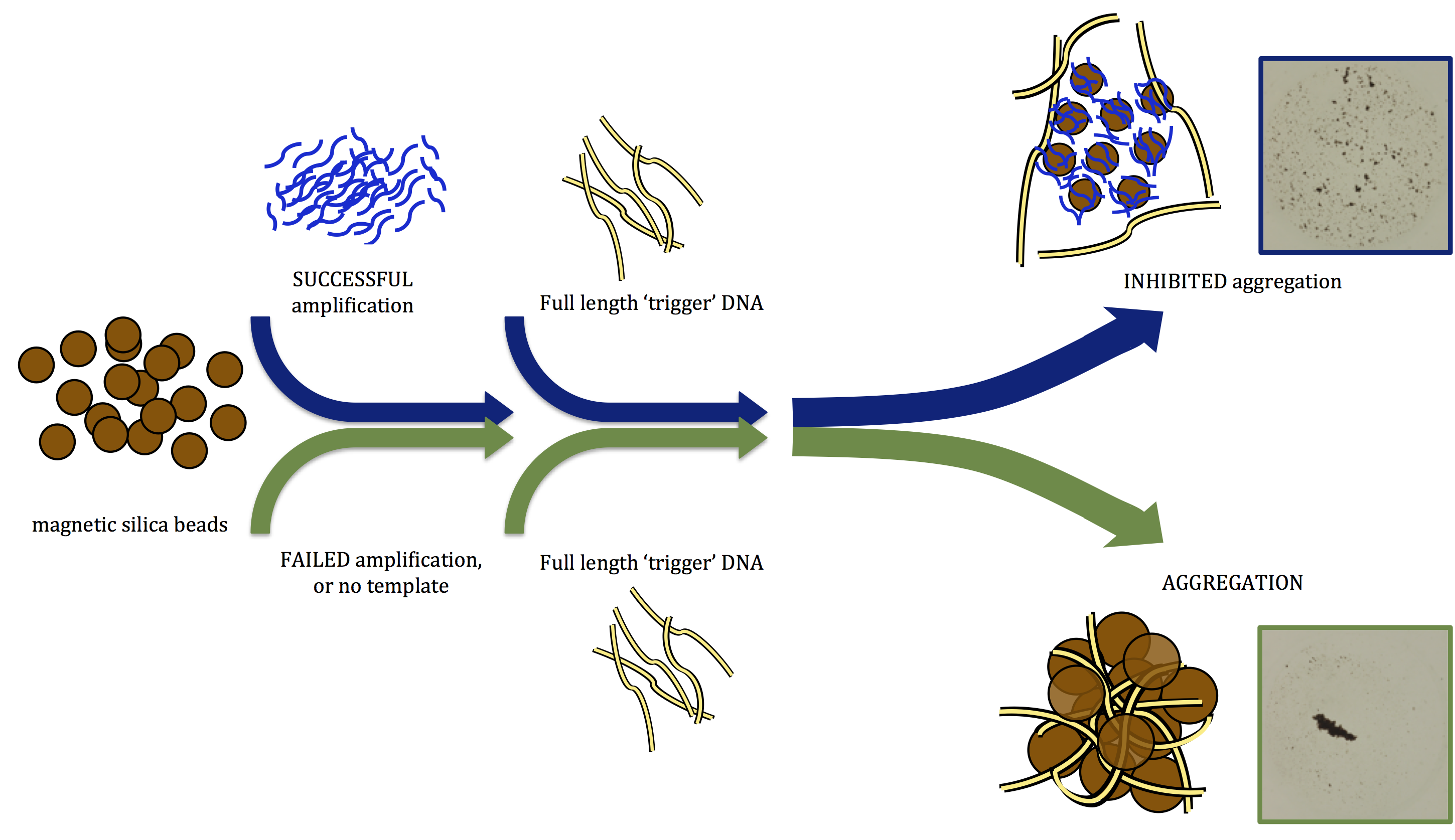

Supplement: S1 Fig — LAMP product is added to a sample of magnetic beads. If the amplification was successful, the presence of short fragments of the target sequence inhibits aggregation by trigger DNA. (TIFF) [file pone.0129830.s001.tiff]

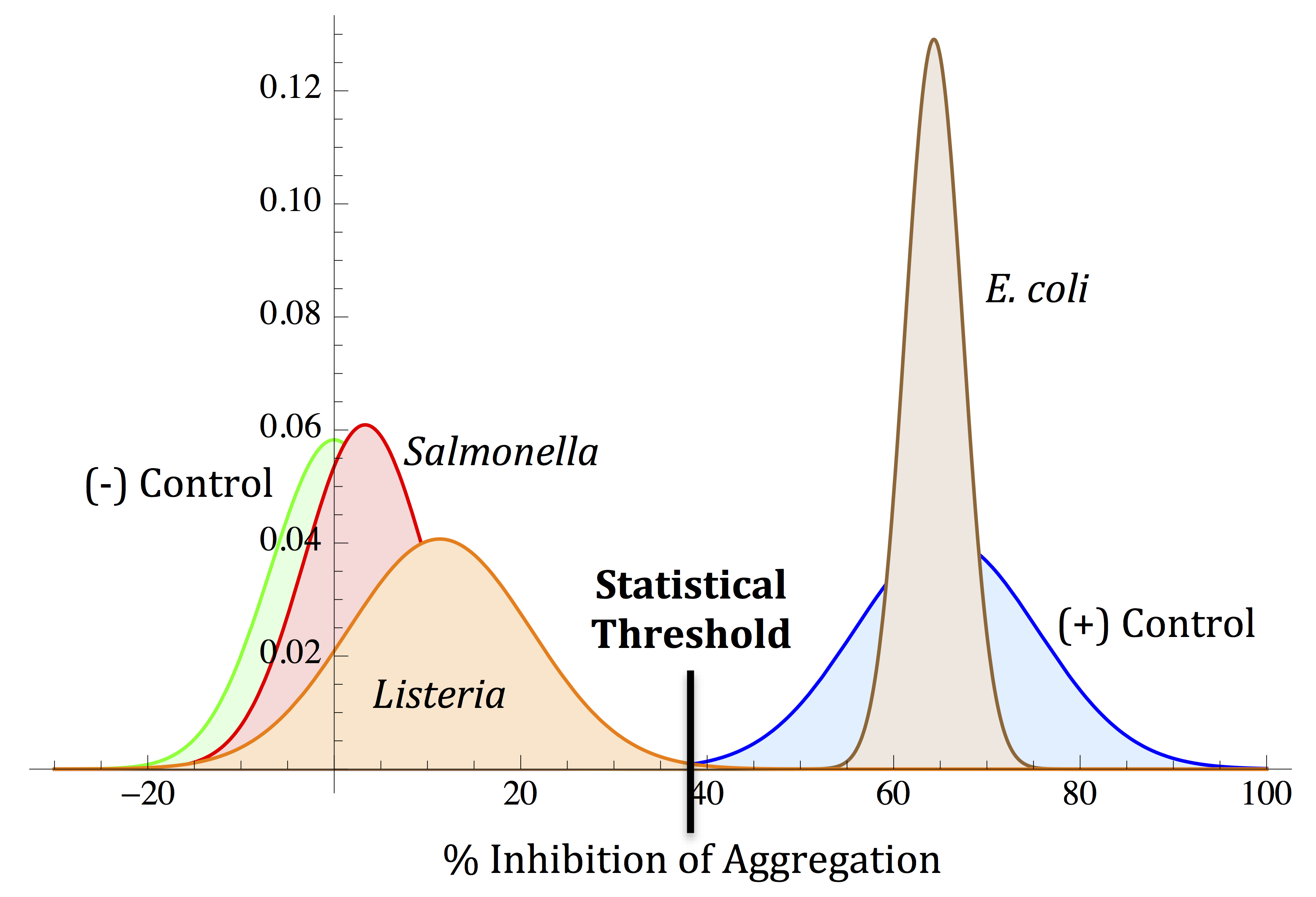

Supplement: S3 Fig — PDFs are plotted for each piece of data (here, E. coli primer specificity) and the statistical threshold is calculated as 3 times the standard deviation of the mean of the positive control. A Mathematica algorithm is used to calculate the probability of any given curve crossing the statistical threshold. (TIFF) [file pone.0129830.s003.tiff]

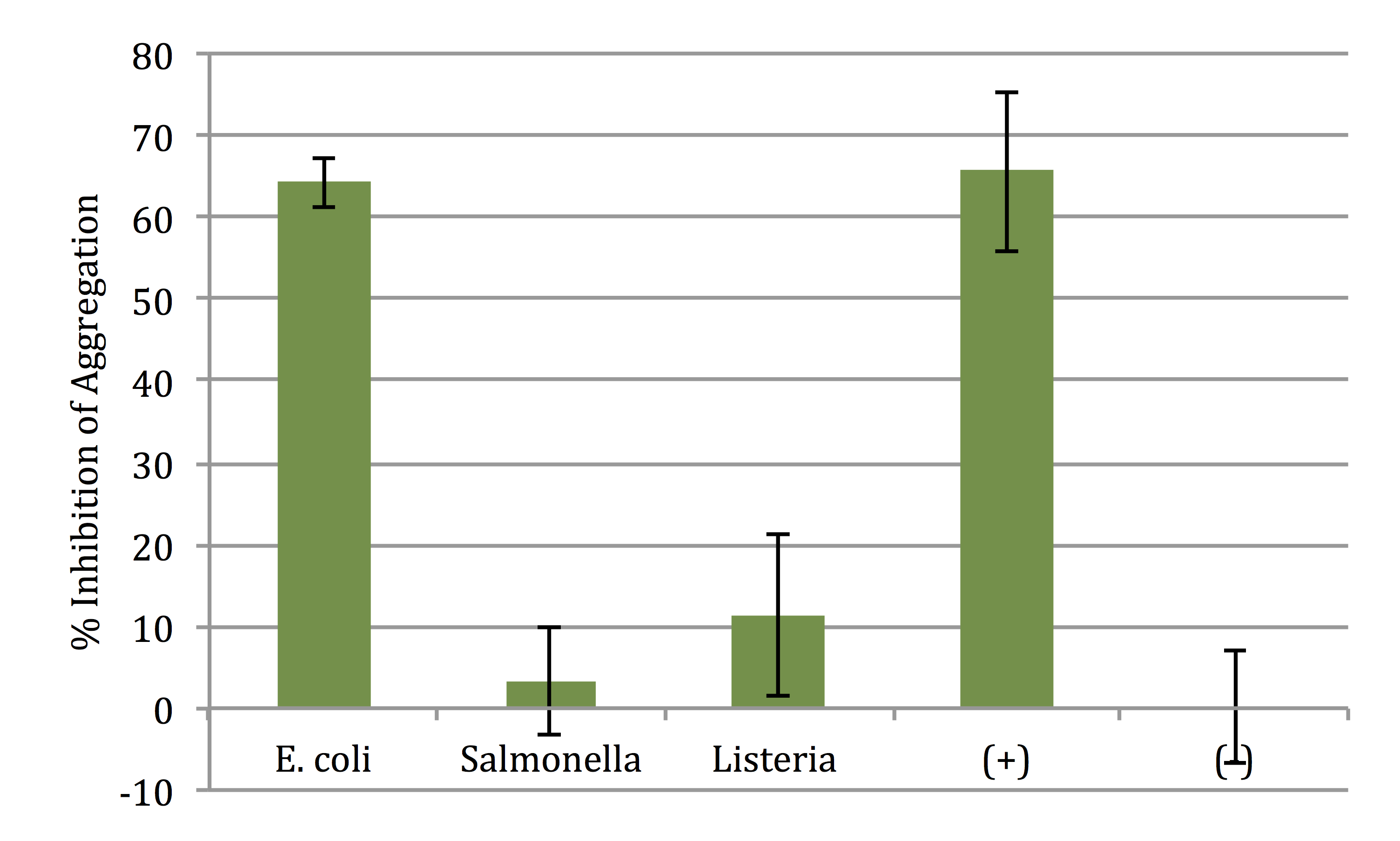

Supplement: S4 Fig — Data from Fig 3A presented as % Inhibition of Aggregation. (TIFF) [file pone.0129830.s004.tiff]

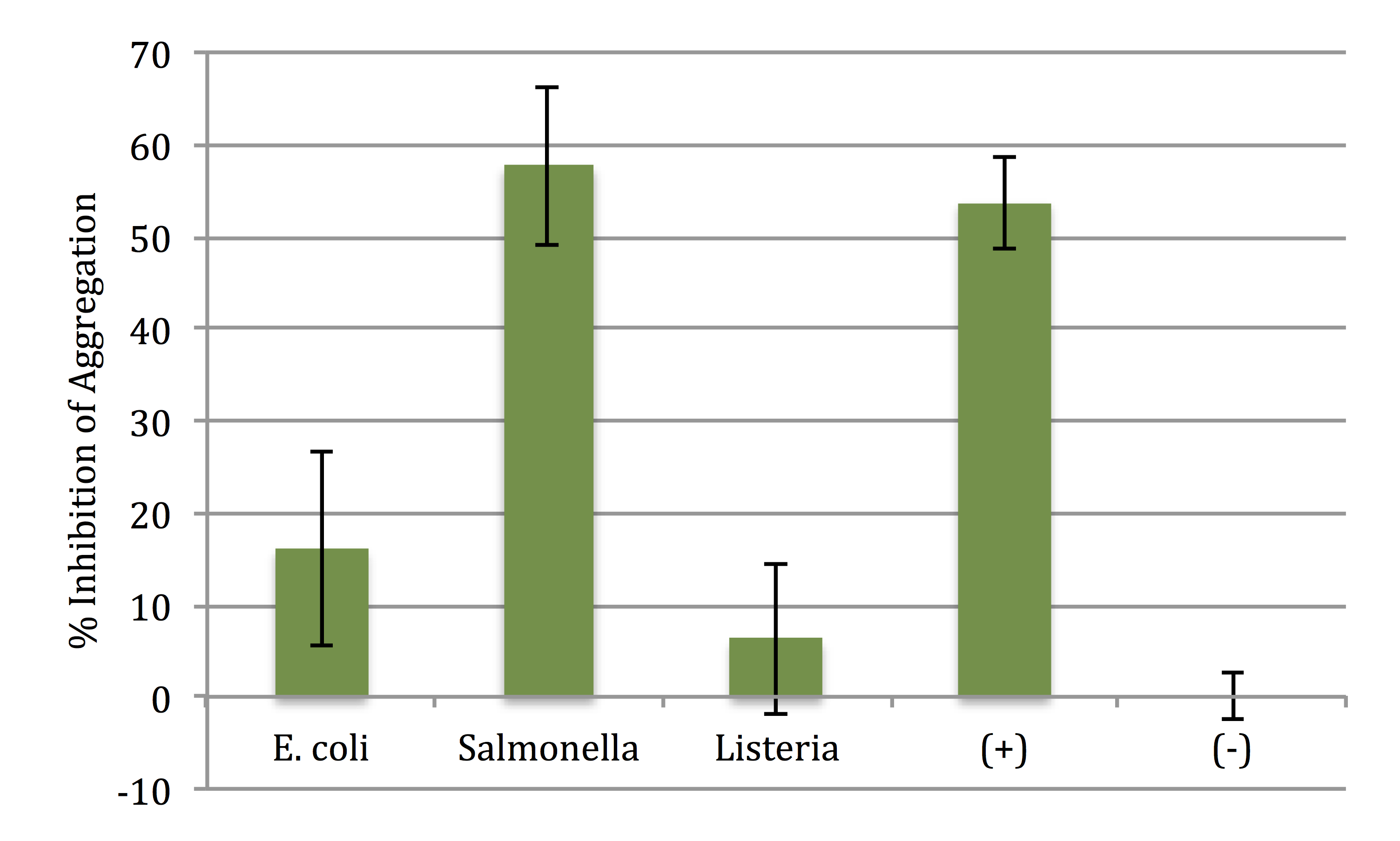

Supplement: S5 Fig — Data from Fig 3B presented as % Inhibition of Aggregation. (TIFF) [file pone.0129830.s005.tiff]

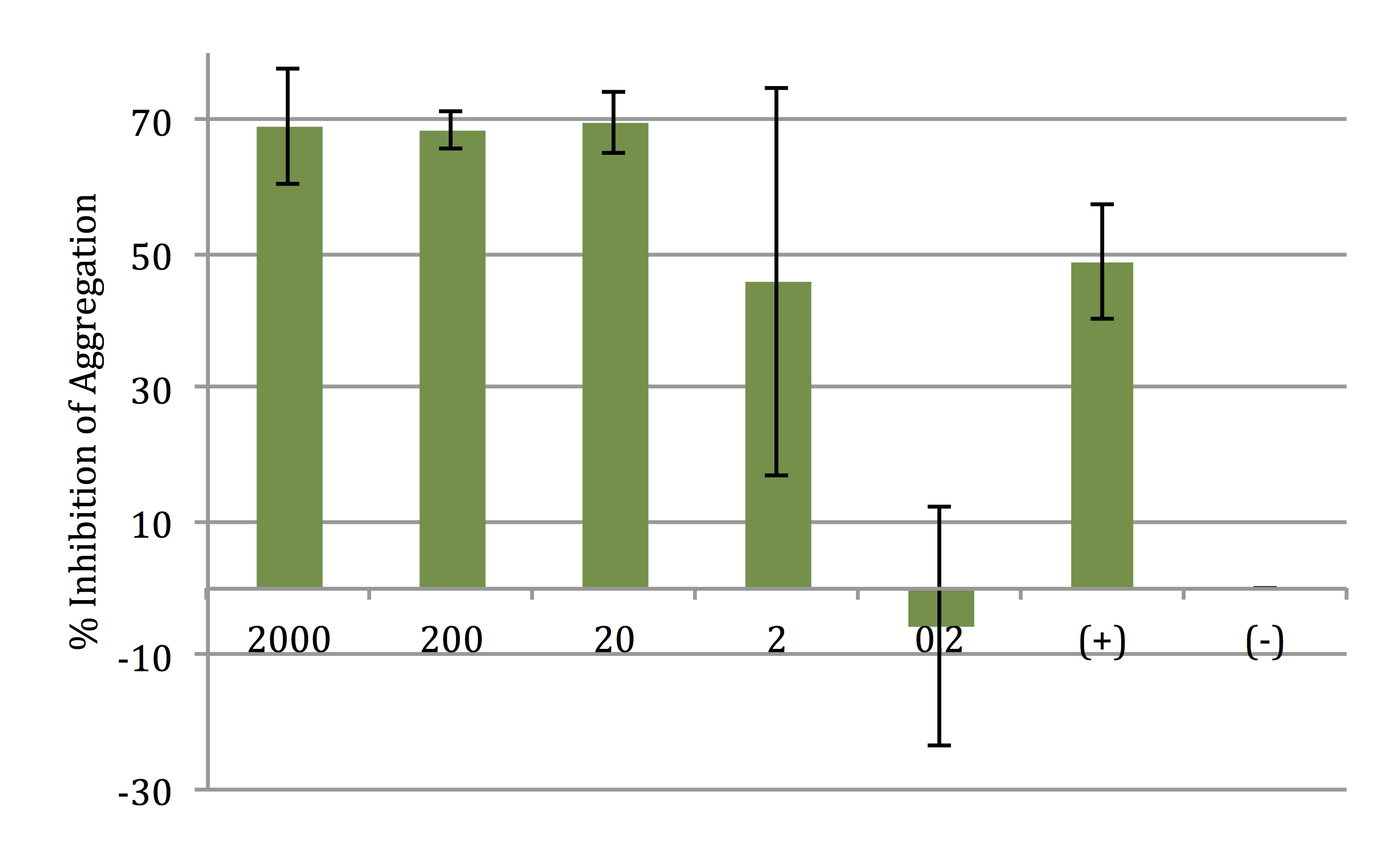

Supplement: S6 Fig — Data from Fig 4 presented as % Inhibition of Aggregation. (TIFF) [file pone.0129830.s006.tiff]

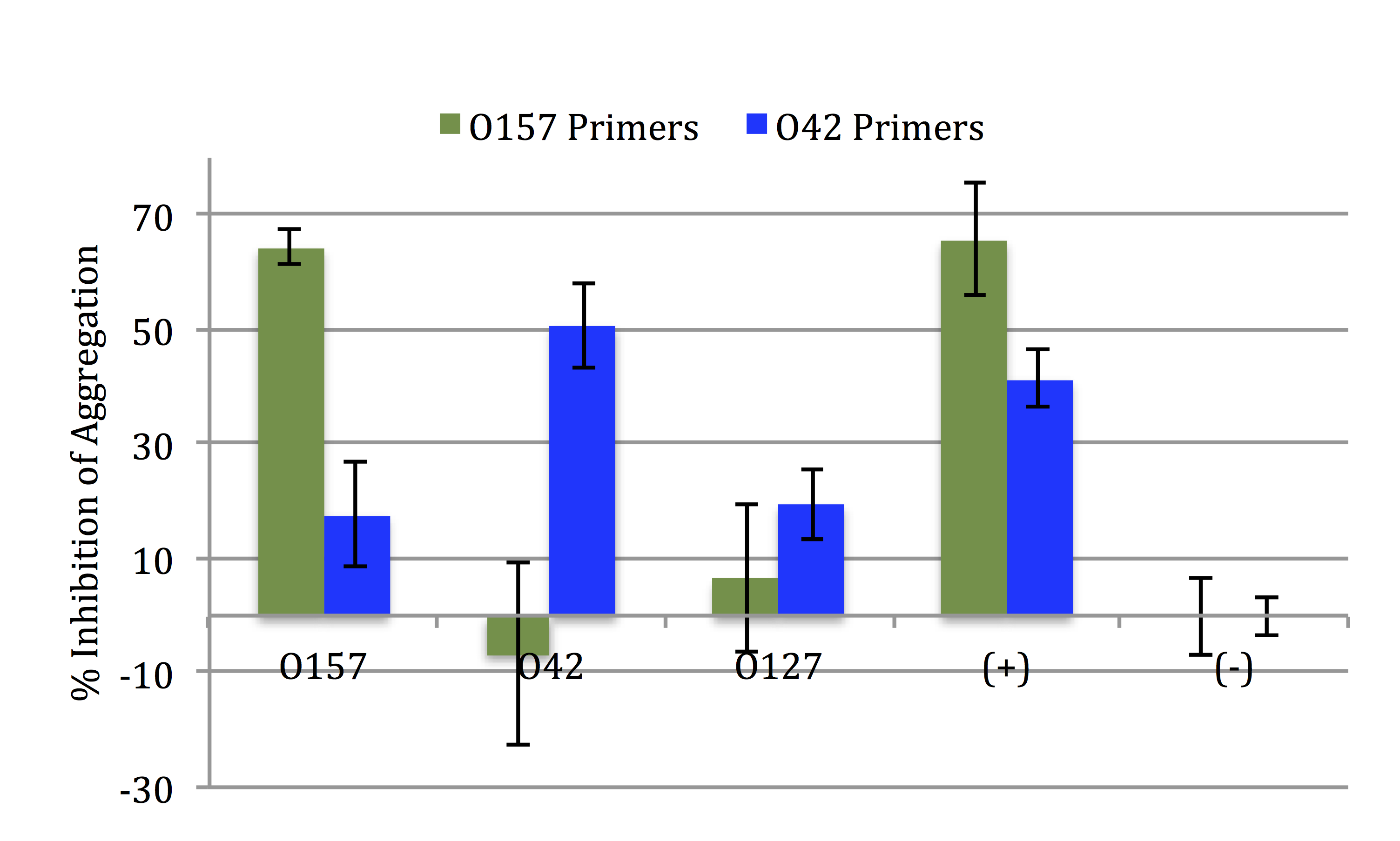

Supplement: S7 Fig — Data from Fig 5A presented as % Inhibition of Aggregation. (TIFF) [file pone.0129830.s007.tiff]

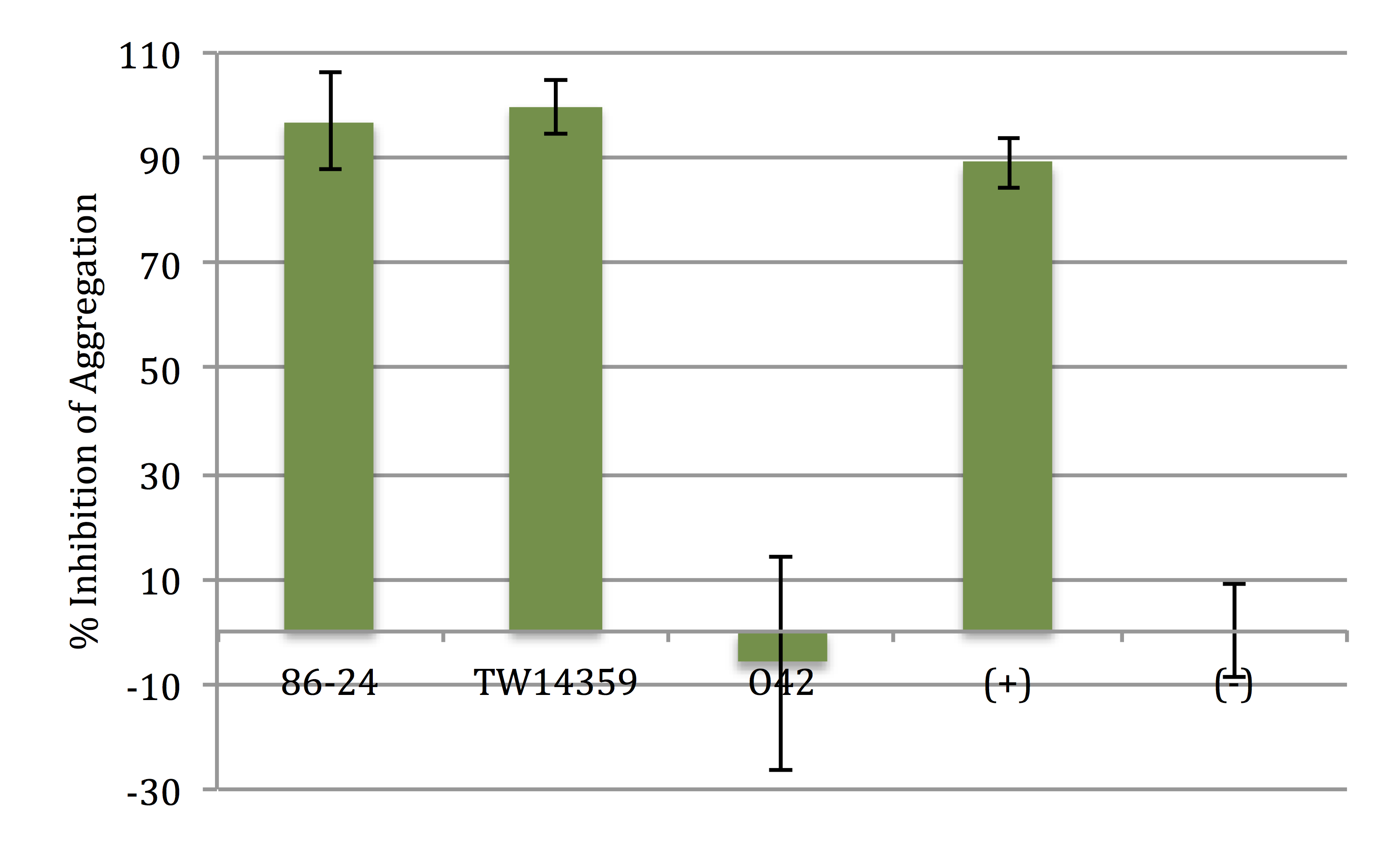

Supplement: S8 Fig — Data from Fig 5B presented as % Inhibition of Aggregation. (TIFF) [file pone.0129830.s008.tiff]

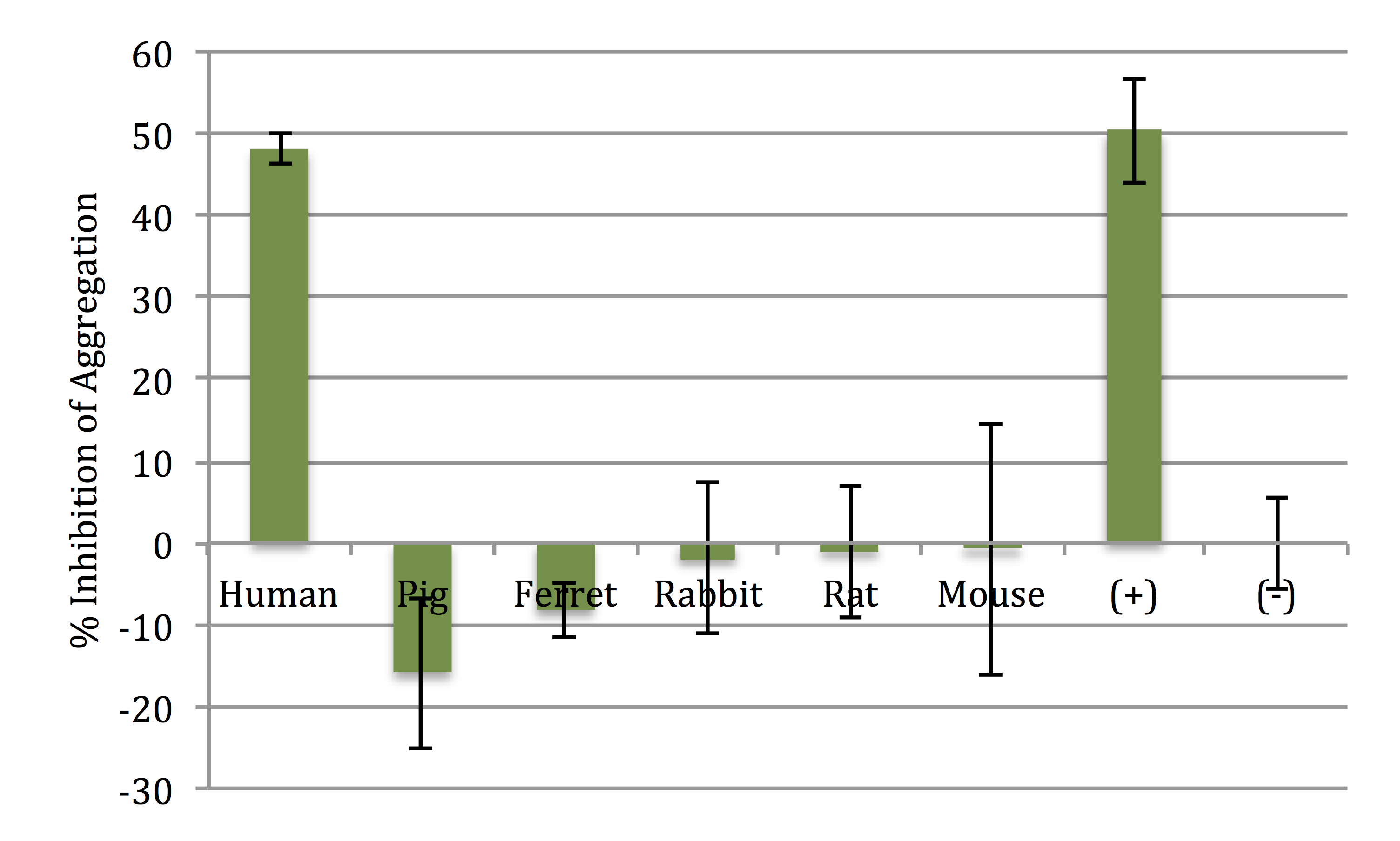

Supplement: S9 Fig — Data from Fig 6A presented as % Inhibition of Aggregation. (TIFF) [file pone.0129830.s009.tiff]

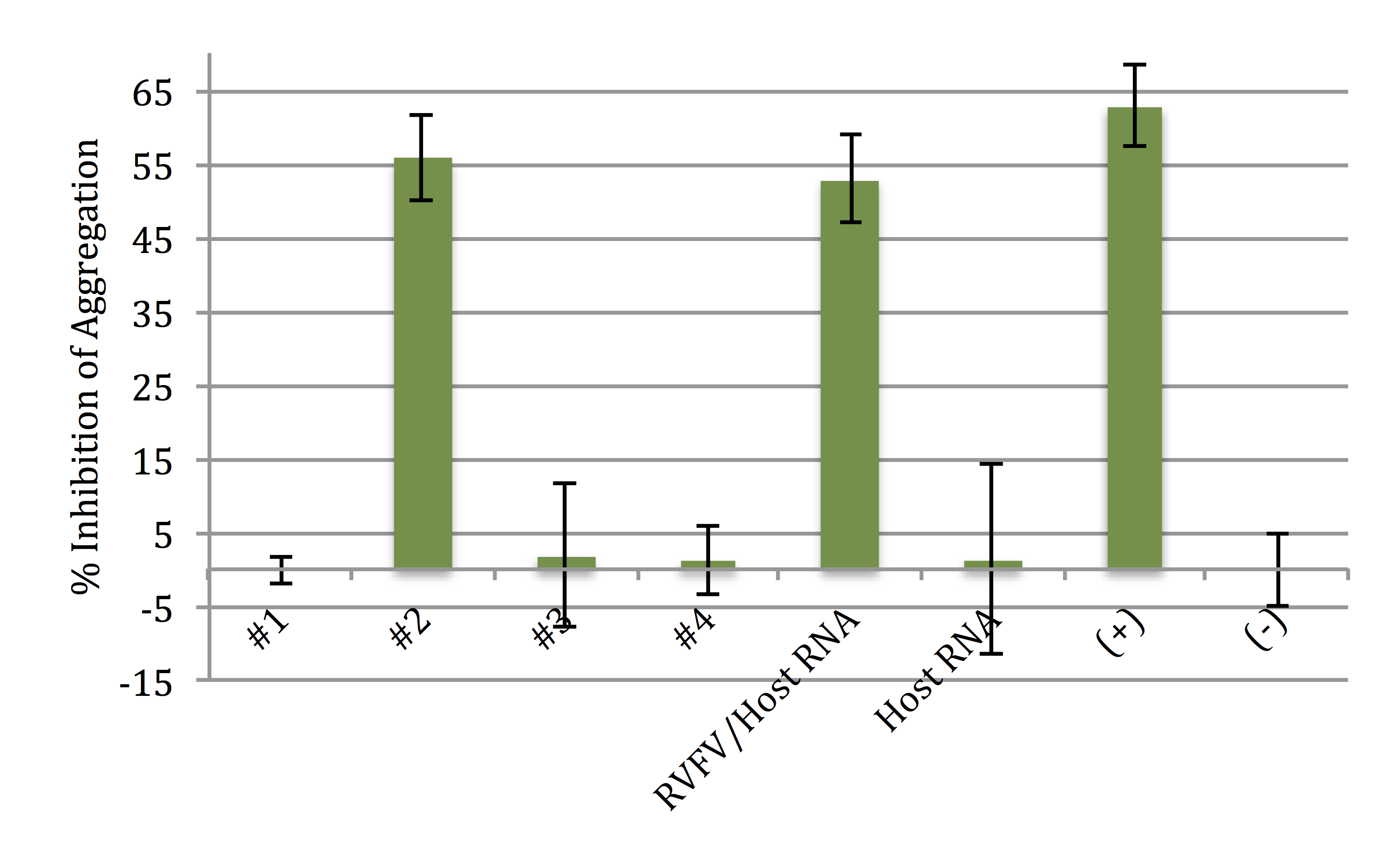

Supplement: S10 Fig — Data from Fig 6B presented as % Inhibition of Aggregation. (TIFF) [file pone.0129830.s010.tiff]

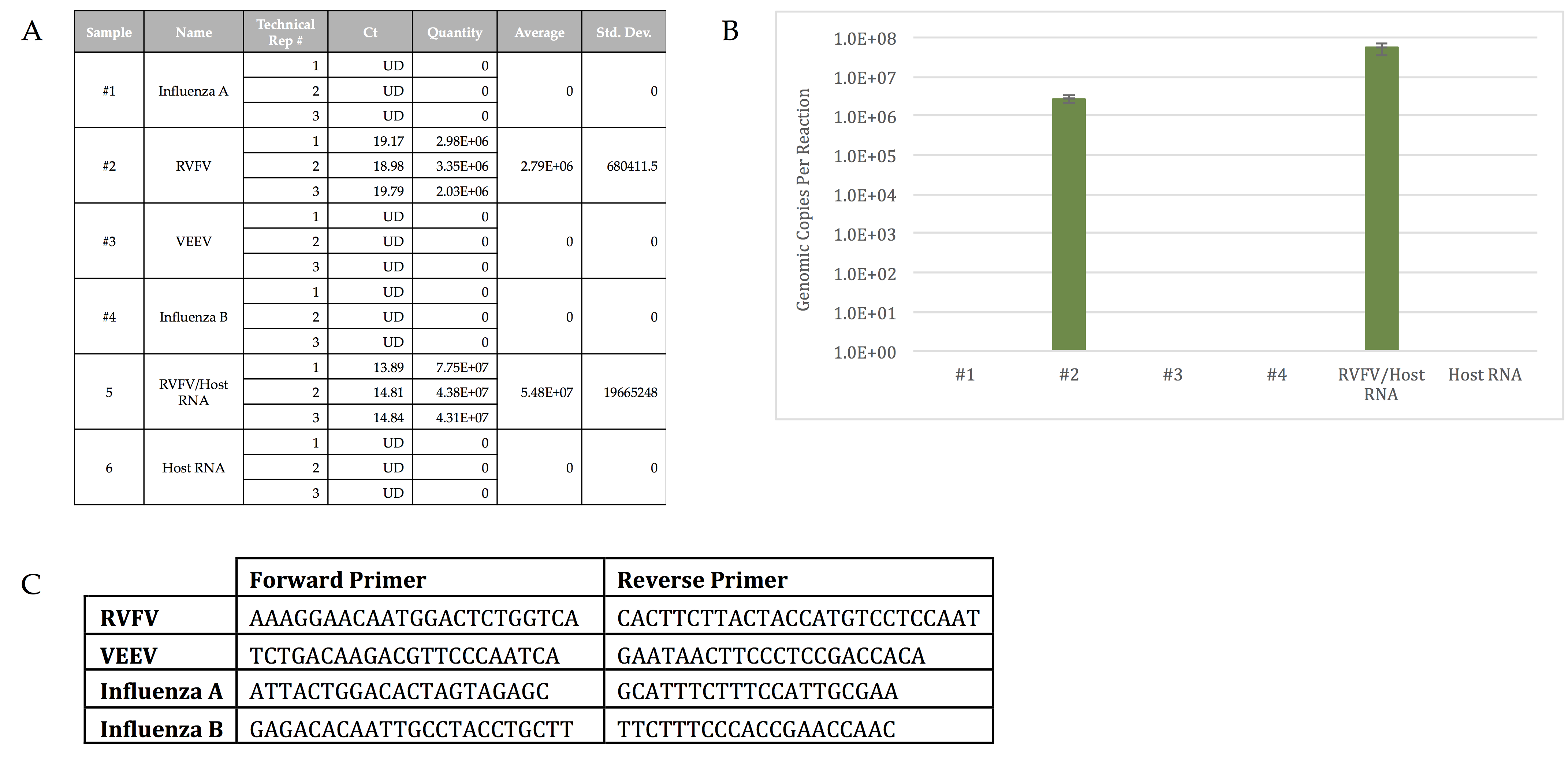

Supplement: S11 Fig — A) Table of results from RVFV qRT-PCR. B) Graph of genomic copies per reaction, confirming blinded viral sample results. C) Primer sequences used. (TIFF) [file pone.0129830.s011.tiff]

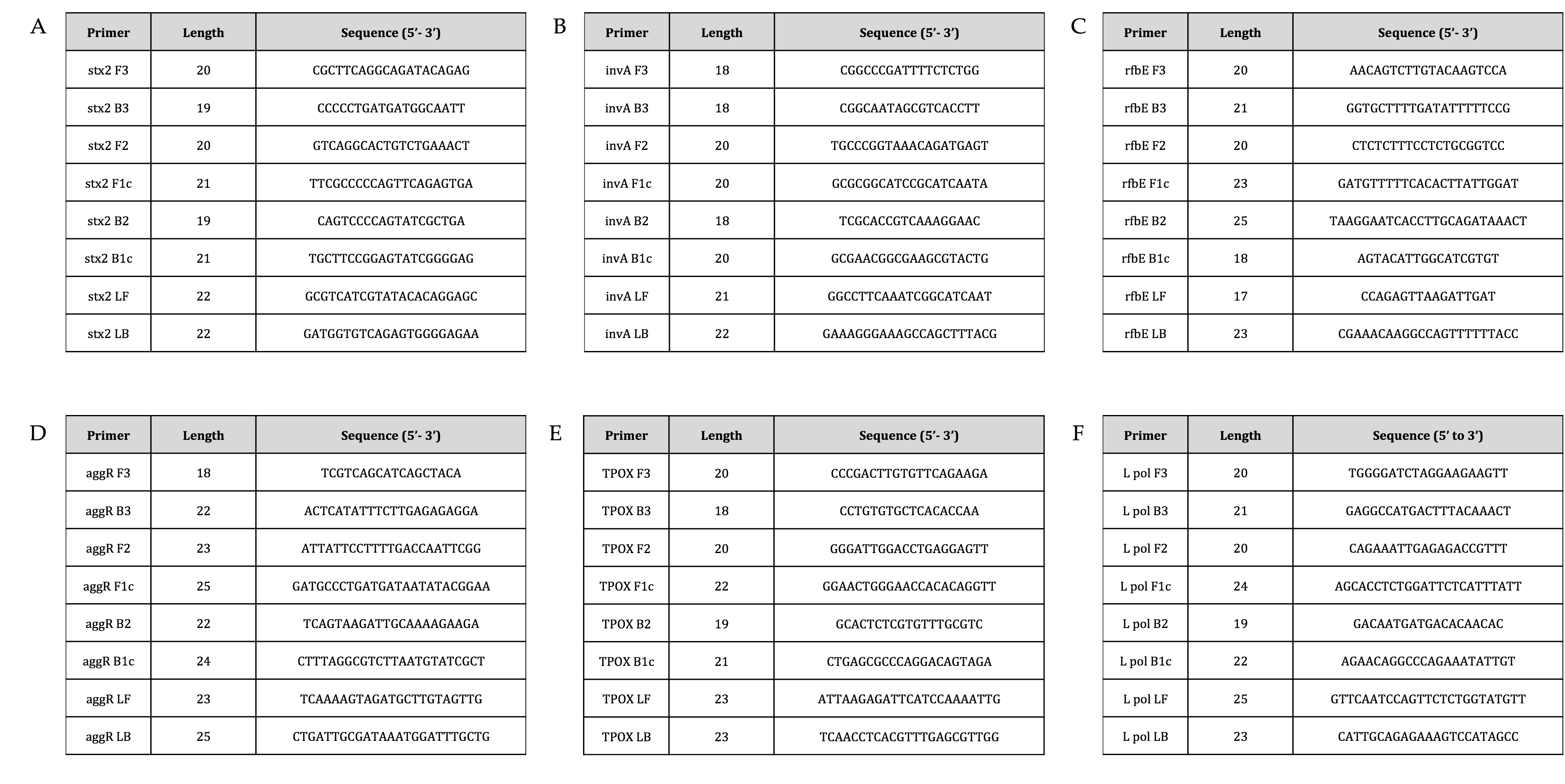

Supplement: S1 Table — A) E. coli stx2, 65°C [33]. B) Salmonella invA, 63°C [34]. C) E. coli O157 rfbE, 65°C [22]. D) E. coli O42 aggR, 65°C. E) Human-specific TPOX, 0062°C. F) RVFV L polymerase, 61°C [35]. (TIFF) [file pone.0129830.s012.tiff]
